# A Hybrid FPGA-Based System for EEG- and EMG-Based Online Movement Prediction

**DOI:** 10.3390/s17071552

**Published:** 2017-07-03

**Authors:** Hendrik Wöhrle, Marc Tabie, Su Kyoung Kim, Frank Kirchner, Elsa Andrea Kirchner

**Affiliations:** 1 DFKI GmbH, Robotics Innovation Center (RIC), Robert-Hooke-Str. 1, D-28359 Bremen, Germany; marc.tabie@dfki.de (M.T.); Su-Kyoung.Kim@dfki.de (S.K.K.); frank.kirchner@dfki.de (F.K.); elsa.kirchner@dfki.de (E.A.K.); 2 Robotics Group, Department of Mathematics and Computer Science, University of Bremen, Robert-Hooke-Str. 1, D-28359 Bremen, Germany

**Keywords:** brain-computer interfaces, mobile computing, embedded systems, fpgas, neuromuscular rehabilitation, movement prediction, embedded brain reading

## Abstract

A current trend in the development of assistive devices for rehabilitation, for example exoskeletons or active orthoses, is to utilize physiological data to enhance their functionality and usability, for example by predicting the patient’s upcoming movements using electroencephalography (EEG) or electromyography (EMG). However, these modalities have different temporal properties and classification accuracies, which results in specific advantages and disadvantages. To use physiological data analysis in rehabilitation devices, the processing should be performed in real-time, guarantee close to natural movement onset support, provide high mobility, and should be performed by miniaturized systems that can be embedded into the rehabilitation device. We present a novel Field Programmable Gate Array (FPGA) -based system for real-time movement prediction using physiological data. Its parallel processing capabilities allows the combination of movement predictions based on EEG and EMG and additionally a P300 detection, which is likely evoked by instructions of the therapist. The system is evaluated in an offline and an online study with twelve healthy subjects in total. We show that it provides a high computational performance and significantly lower power consumption in comparison to a standard PC. Furthermore, despite the usage of fixed-point computations, the proposed system achieves a classification accuracy similar to systems with double precision floating-point precision.

## 1. Introduction

In the last couple of years, the analysis of physiological data has been successfully applied to achieve different research or application goals, e.g., to control different kinds of devices, to allow locked-in patients to communicate with their environment, or to adapt technical devices to the cognitive state of a human [1,2,3,4,5,6,7,8,9,10,11].

Especially in the field of neurorehabilitation and support of daily activities the interest to use physiological data has increased. A restriction or loss of the mobility due to a neurological disease or injury, such as stroke or spinal cord injuries, usually reduces the patients quality of life considerably. In such a case, physiological data can be applied to adapt a rehabilitation or support device, for instance an active orthosis or exoskeleton, in order to accelerate or improve the rehabilitation process of patients or to provide a suitable amount of support in daily life.

In this context, the capabilities of the rehabilitation device are a relevant issue. A rehabilitation device should support therapy approaches that involve functional training that is tailored to the physical conditions and therapy state of the patient (assist-as needed) [12,13].

To achieve this, the adaptation of robotic rehabilitation systems (e.g., active orthoses or exoskeletons) [14,15,16,17,18,19,20,21,22] with real-time analysis of physiological data related to neuronal or muscular activity to predict movements seems to be a promising approach [23,24,25,26,27,28,29,30]. Data that can be acquired non-invasively from the human body, for instance surface Electroencephalography (EEG) and surface Electromyography (EMG), have a range of advantages for practical purposes, e.g., they provide a good time resolution, the data acquisition equipment is relatively cheap and portable. Several studies could show that the approach to adapt assistive or therapeutic devices with respect to physiological data motivates the patients and encourages the active participation in the exercises, which can result in an increased neuroplasticity in, e.g., stroke rehabilitation and, thus, therapy success [31,32,33,34,35,36].

One important aspect to achieve practical usability of a therapeutic device is to provide a high mobility [37,38]. This can be achieved by the development of small computing systems that control the device, but also process the physiological data and are embedded into the rehabilitation device or other equipment like wheelchairs [39] and exoskeletons [29]. However, systems like Brain-Computer Interface (BCI) are nowadays often used in artificial and stationary experimental setups. Reasons for this are (1) the immobility of the utilized hardware for data acquisition, (2) the experimental conditions in which BCI systems are still employed, but also (3) the size of the computing devices required for the analysis of the human EEG or EMG. Recently, substantial progress has been achieved regarding (1) and (2) (see Section 2.4). Although the development of highly performant and power-efficient miniaturized computing devices is an important aspect for rehabilitation applications, aspect (3) has rarely been in the focus of previous research.

For many rehabilitation applications, four different types of requirements are important for the development of embedded systems. First, the system should be as small as possible and have a low power consumption. Secondly, a computing device that is embedded into a larger rehabilitation device must provide the ability to communicate with various other systems and provide additional features such as the possibility to record data. These “high-level” functionalities are very diverse in structure and their implementation is usually based on a wide range of different software libraries. Fortunately, apart from certain types of communication (e.g., motor control to actuate a device), these functionalities are mostly not time-critical. Hence, these features are well-suited for a realization in software. and can be performed by a generic Central Processing Unit (CPU). Thirdly, for the actual processing of the physiological data, a set of different signal processing and Machine Learning (ML) algorithms has to be applied on the incoming stream of physiological data in real-time. Depending on the amount of data and the complexity of the algorithms, this part can be computationally expensive. If we aim to use movement prediction for the control of an assistive or therapeutic device physiological signals must be chosen that provide this information. However, their analysis should be performed with minimal latency. Fourthly, if different physiological signals are combined to predict movements in a hybrid approach, multiple different signal processing operations have to be applied asynchronously and in parallel to different streams of data. This can be a problem if the processing should be performed in miniaturized processing devices.

In this paper, we propose a hybrid Field Programmable Gate Array (FPGA)-based approach for the implementation of a hybrid BCI, since this can fulfill the requirements discussed above. Our system is based on the Xilinx Zynq^®^ [40], that combines a generic CPU with programmable logic (which is equivalent to an FPGA). This combination makes it possible to implement high-performance processing devices, which are power-efficient, have small dimensions, and can replace a bulky stationary PC. The CPU of the system offers the possibility to use standard software for user interaction as well as high-level and configuration tasks, which are tasks that are usually not performance-critical. Computationally expensive and time-critical operations can be transferred to hardware-accelerators that are implemented using the FPGA, which assists the generic CPU. Multiple hardware accelerators can work independently and asynchronously on different tasks, e.g., to process multiple physiological signals at the same time. Since FPGAs offer the capability to exploit massive parallelism to accelerate computations, they represent a well-suited technology for the implementation of performance-critical operations. FPGAs achieve this by offering the possibility to use computing architectures that can represent the underlying algorithms. For instance, in signal processing and machine learning systems such as BCIs, data is often processed by multiple consecutive operations. For these applications, the system can be represented by dataflow architectures, where data is streamed directly from one processing step to the next one. This allows exploiting pipelining parallelism and avoiding overhead due to, e.g., transfers between hardware accelerator and external memory. For the target application, a further advantage of FPGAs is the possibility to obtain a tight integration of the actuated rehabilitation device and the data processing system, since FPGAs offer the possibility combine motor control and commutation methods with data processing operations on the same die. Furthermore, an advantage of an FPGAs over other types of hardware-accelerators (e.g., hard-wired Application-Specific Integrated Circuits (ASICs)) is the possibility to benefit from hardware acceleration, but to still have the possibility to adapt the system to different application properties, for instance to the number of different channels used by a specific system, or to exchange or add specific components, which can be helpful if better methods become available or the application requirements change.

In many applications, it is helpful to rely on multiple physiological signals instead on a single one [41]. Since different physiological signals exhibit different properties and contain different information, it can be advantageous to switch between them or use a combination of them. Especially in robotic devices for neurorehabilitation, the combination of EEG and EMG provides advantages over a single modality. For instance, EEG and EMG can be combined to either predict as many movements as possible or to enhance the reliability of movement prediction. If the system provides the capability to combine EEG or EMG-based movement predictions in different ways, it can be used to adapt a possible rehabilitation system to the diagnosis of a patient or different needs of patients in different stages of therapy [25,42]. Depending on the type of combination, the system can be used in therapy sessions for successfully treated patients to enforce their involvement, or alternatively in therapy sessions for patients who still have great difficulties to initiate or execute movements or control the rehabilitation device by means of physiological data. EEG and EMG also exhibit different temporal properties in relation to a movement onset, i.e., it is possible to detect movement intentions early using EEG, while changes in the EMG can only be observed close to the actual movement onset. Moreover, the feasibility to predict movements using EMG might depend on the severity of a paresis, is not applicable for paralyzed patients, and is difficult for patients with spasms or tremor. Furthermore, the combination can help to enhance the reliability of control commands of the device [43,44]. A further advantage of such a combination is the possibility to *adapt* the data-dependent signal processing and machine learning methods used for the EEG-based movement detection by using the EMG-based predictions [45,46].

### Contributions and Structure of the Paper

This paper makes two major contributions. The first contribution is the development of an FPGA-accelerated mobile system developed for the prediction of upcoming movements. The system is based on a self-developed hardware platform, the ZynqBrain (see Section 3.1), and provides the possibility to combine standard software for high-level tasks and user interaction with application-specific hardware accelerators that increase the computing performance of the overall system. The signal processing related parts of the proposed system are real-time compliant and provide a high amount of parallelism. The proposed system is validated using both an offline evaluation that is making use of former recorded data and an online evaluation during which physiological data is recorded and processed continuously. We show that the system outperforms a standard PC regarding processing speed, while providing an equivalent classification accuracy. Moreover, the system has a low power consumption and a small form factor. This enables the development of future embedded or wearable rehabilitation systems that require a tight connection between signal processing and control of the rehabilitation device.

In the second contribution, we show that the parallel processing capabilities of our proposed mobile system can be used to process multiple different streams of data at the same time in order to increase the reliability of the movement predictions. This is achieved by combining movement-related patterns with each other or with EEG patterns evoked by task-relevant signals, such as an instruction of the therapist to the patient. This is of special interest for patients in early stages of therapy that require instruction and show poor movement capabilities. We expect that the so called P300 is evoked by instructions given by the therapist to the patient. We showed already that this pattern is evoked by seldom, task relevant instructions and can be recorded in complex interaction scenarios [47]. In this work we use a more controlled scenario, where we combine an oddball paradigm with delayed responses on task-relevant events to evoke EEG patterns related to the recognition of task relevant stimuli and movement preparation. Here, we investigate in a controlled scenario whether single-trial P300 detection can be used to improve movement classification performance for controlled therapy sessions in which patients perform movements only after instruction of a therapist. For this purpose we systematically combine the analysis of EMG-data and the two different EEG-patterns to predict movements before they occur and to evaluate the effect of different combinations.

The remainder of this paper is structured as follows. In Section 2, we give an overview about related work. Subsequently, the architecture of the proposed system and the applied data processing methodology are discussed in Section 3 and Section 4, respectively. The proposed system is evaluated on physiological data obtained in an example application scenario, the procedures for the experimental evaluation are described in Section 5. The obtained results are presented and discussed in Section 6. The conclusion and a plan for future work is given in Section 7.

## 2. Related Work

### 2.1. EEG-Based Movement Prediction

Electroencephalography (EEG) data can be analyzed to detect neural activity related to movement intentions or upcoming movements, such as Event-Related Desynchronization/Synchronization (ERD/ERS) or Movement Related Cortical Potentials (MRCPs) [48,49,50]. ERD/ERS reflects changes in the *oscillatory* activity of neural networks by an attenuation or increase in the power of specific frequency bands [51,52]. MRCPs are small changes in the *amplitude* of EEG related to movement planning and execution [53,54,55]. EEG-based movement prediction is especially helpful in assistive devices for telemanipulation [56,57] and rehabilitation [12,25,58]. Predicting movements using an MRCP-based approach has the advantage that upcoming movements can be detected very early [55]. However, due to the low Signal-to-Noise Ratio (SNR) of raw EEG data, a range of conceptually very different and computationally expensive algorithms has to be applied to extract useful information [59,60,61]. Hence, the utilized computing hardware must guarantee a sufficiently high performance and low latency to preserve earliness of prediction.

### 2.2. EMG-Based Movement Prediction

The Electromyography (EMG) measures electrical potentials generated by active muscles. Usually surface EMG is relevant for clinical applications and physiological computing [62,63]. The electrodes measure the summed activity of several muscle fibers. EMG analysis has been successfully applied to detect or predict movements [64] for a range of different applications, such as active orthosis and prosthesis or exoskeleton control [65,66,67]. However, the usage of EMG-based movement prediction is not always suitable, e.g., for paralyzed patients caused by spinal cord injuries.

### 2.3. Hybrid BCI Systems

The focus of most research on EEG-based BCIs is to provide basic communication or control to people with severe motor impairments [1,68,69]. The development of novel augmented BCIs, i.e., BCIs that can be used by individuals in everyday applications may enhance the way how people interact with technology in their everyday environment [70]. Especially using BCIs for the control of neurorehabilitation devices or orthoses and prostheses [71,72,73,74,75] receives increasing attention. In this context, enhancing the applicability or reliability of BCI systems by combining multiple BCIs to create hybrid BCIs represents an important aspect [41,76,77].

A signal frequently used in such applications is the P300-Event-Related Potential (ERP) [47,78,79]. For instance, P300-based BCIs have been successfully combined with motor imaginary-based BCIs to enhance the control possibilities [72,80] or reliability [81] of the combined system. Hence, hybrid BCI systems can avoid wrong predictions of the upcoming movements to decrease frustration of the patient and increase therapy success. Especially, movement prediction combined with P300 detection can ensure a robust prediction of the upcoming movements with an increased prediction accuracy, since the P300 is detected with a higher accuracy compared to movement prediction. A further possibility is to combine an EEG-based BCI-system with EMG signals, which can similarly increase the reliability of the combined system [25,42,43,82,83,84,85,86,87]. However, to combine several modalities (e.g., EEG, EMG), it is important that the utilized computing system is capable to process several different data streams in parallel and real-time to be able to combine the individual predictions. Another possibility that we do not consider in this study is to increase the reliability of movement predictions by combining the predictions with other modalities, e.g., eye gaze or limb position [84,88].

### 2.4. Mobile and Embedded BCI Systems

Recent progress in the development of portable EEG systems and dry electrodes [89,90,91,92,93,94] improves aspects (1) and (2) as mentioned in Section 1. For example, to process the data acquired by the acquisition hardware, most systems use wireless connections such as Bluetooth and IEEE 802.15.4 Zigbee or RF transmission [95,96,97,98,99,100,101] to transfer the data to a separate processing device, stationary PC or laptop. However, this might generate additional latency and increase power consumption. Further, lossy compression to reduce the data rate can cause artifacts [102,103]. These are critical aspects especially for real-time movement prediction.

Hence, it would be beneficial to use devices which allow a tight integration of data acquisition hardware, computing device and rehabilitation equipment. In order to integrate computing devices into other systems such as orthosis or exoskeletons, they require small physical dimensions, low weight and low power consumption (in order to be able to use small accumulators) [104]. However, since the employed signal processing operations for the detection of specific EEG patterns are often computationally expensive, current mobile [105,106], and FPGA-based systems [107,108,109,110,111] BCI systems often rely on a small number of electrodes, which is advantageous for practical reasons and sufficient for *simple* approaches based on, e.g., the detection of Steady-State Visual Evoked Potential (SSVEP) [105,112] or seizures [113]. However, for real-time movement prediction or hybrid approaches, the ability of the system to process high-dimensional data is a relevant issue in the *single trial analysis* of EEG and EMG. This is in particular the case when the detection of specific EEG/EMG patterns is difficult and the data is affected by noise or artifacts, as it likely is the case in complex rehabilitation applications [11]. In this case, the usage of a high number of channels is beneficial for accurate classification, since it allows to apply spatial filter-based artifact attenuation [37]. Accordingly, specialized signal-processing systems that use complex algorithms and apply these in an online-fashion and real-time are needed. A recent trend ist the development of such systems for, e.g., fall prediction [110].

Resulting from the discussion above it is clear that real-time analytics of physiological data for rehabilitation applications places high methodological and technological demands on devices that should be used to achieve this: the dimensionality of the data is high, the SNR is low, and complex algorithms have to be used to extract useful information from the data. Furthermore, if the BCI system is hybrid, different streams of data have to be processed independently and in parallel. Finally, the applications might underly strict real-time constrains. In order to solve this problem, high performance embedded computing systems are required that can employ a range of different signal processing and ML techniques to process the physiological data in real-time and have a low power consumption.

### 2.5. Field Programmable Gate Arrays

FPGAs are a technology that provide the possibility to build parallel architectures that process large amounts of data efficiently under hard real-time constraints and to perform multiple tasks at the same time. Hence, they are well-suited for the intended application of real-time movement prediction.

FPGAs consist of flexible programmable logic elements that contain Look-Up Tables (LUTs) and Flip Flops (FFs) to realize complex combinational logic functions and register-transfer logic. With the increasing technological capabilities, FPGAs became a popular alternative to CPUs and Graphics Processing Units (GPUs) for different embedded and high performance computing tasks [114,115,116,117,118,119]. To support these kinds of tasks, FPGAs contain different kinds of supporting hardware primitives that can be utilized for these operations, such as fast accessible memories (in terms of Block Random Access Memorys (BRAMs) [120]) to store intermediate data, and Digital Signal Processing (DSP) slices (e.g., the DSP48 slice in Xilinx FPGAS [121]) to support Multiply ACcumulate (MAC) operations.

Since many DSP and ML algorithms require primarily multiplications and additions but no complex control logic, FPGAs are a suitable candidate solution for their implementation. A development that became increasingly popular in recent years is the combination of programmable logic with CPU cores that allow to build System-on-Chips (SoCs) such as the Xilinx Zynq^®^ [40] or Altera Stratix^®^, Arria^®^ and Cyclone SoCs^®^ [122]. These devices allow to combine standard software executed on the CPU with dedicated hardware accelerators implemented in programmable logic.

However, the development complexity of FPGA-based systems is significantly higher than the usage of a standard software library such as pySPACE [123] or OpenViBE [124]. A current trend to simplify the development process is High-Level-Synthesis (HLS), i.e., the usage of high-level languages such as C or OpenCL instead of the conventional hardware description languages [125,126]. Although this reduces the development complexity considerably, it has been found that designs obtained by using HLS often have a lower performance and higher resource utilization than customized hardware accelerators [127]. Hence, a possible solution is to provide a framework that contains a set of flexible IP cores that can be adapted to specific application requirements and can be easily integrated into larger SoC designs.

A further problem with FPGAs is that floating-point arithmetic is expensive in terms of resource utilization. Hence, usually fixed-point arithmetic is used as a replacement. However, fixed-point arithmetic provides a smaller dynamic range for the representation of values which can result in numerical problems or a decrease of the accuracy of the obtained results. Hence, it is important to evaluate whether the fixed-point arithmetic leads to a decrease of the classification performance.

### 2.6. Dataflow Architectures and Hardware Acceleration

The parallel processing capabilities of an FPGA can only be exploited if they are used to implement a suitable processing architecture. A paradigm that can implement architectures that achieve this and fit especially well to the structure of signal processing systems is the dataflow computing paradigm [128,129,130,131,132]. In this paradigm, independently and asynchronously operating actors exchange data via dedicated channels. If a sufficient amount of input data is available on the input channels, the execution of an actor is enabled. Subsequently, the actor consumes data from the input channels, processes it, and elicits the products of the computation on its output channels. The asynchronous execution of multiple actors makes it possible to design signal processing systems that utilize a large number of parallel operating Processing Elements (PEs). Hence, this computing paradigm is an approach well-suited to map signal processing and machine learning applications to FPGAs [133,134,135].

Dataflow models differ in expressivity and analyzability. A high analyzability is especially important for for safety-critical applications as it is the case in medical and rehabilitation applications. Hence, our system is based on the self-timed Synchronous Dataflow (SDF) model [129]. This model requires no complex scheduling algorithms to coordinate the work of the actors and can guarantee deadlock-freeness and bounded memory usage. However, SDF-based systems are not Turing equivalent, i.e., it is not possible to use an SDF-based system to run ordinary software.

The actors can be of different granularity, ranging from fine-grained actors that implement single arithmetic operations up to coarse-grain actors that implement a complete algorithm and include control-flow instances or finite state machines as long as they obey dataflow behavior. A further possibility is to combine standard-hardware architectures, like von Neumann-based CPUs, with dedicated hardware accelerators that follow the dataflow enhance the computing performance of specific, time-critical operations [136]. Accordingly, dataflow architectures are a promising approach to build high-performance and real-time compliant embedded systems.

## 3. Hard- and Software Architecture of the Hybid System

To achieve the constraints related to processing performance, power consumption and physical size, but also provide a high usability, our system uses a combined CPU/FPGA solution. Choosing such an embedded two-in-one solution permits the usage of standard operating system like Linux and a wide range of software on the CPU (see Section 3.3), but provides also the possibility to accelerate specific computations using hardware accelerators implemented in the FPGA.

The basic building block of the system is the self-developed printed circuit board ZynqBrain (Section 3.1). The FPGA-based processing architecture consists of a set of application-specific hardware accelerators realized via the dataflow computing paradigm (Section 3.2). To provide a customary usability of the system, we use a software layer based on pySPACE (Section 3.3).

A further advantage of such a combined CPU/FPGA-based approach is the possibility to establish a direct interface between the components used for biosignal processing and a rehabilitation device. This makes it possible to incorporate the outputs of the movement prediction system directly in the control algorithms of the rehabilitation device. However, in the current study the main focus is to evaluate the accuracy, performance, and power consumption of the developed system and evaluate the feasibility of such an approach for future applications in robotic rehabilitation devices.

### 3.1. The ZynqBrain Electronics Platform

**Figure 1:**
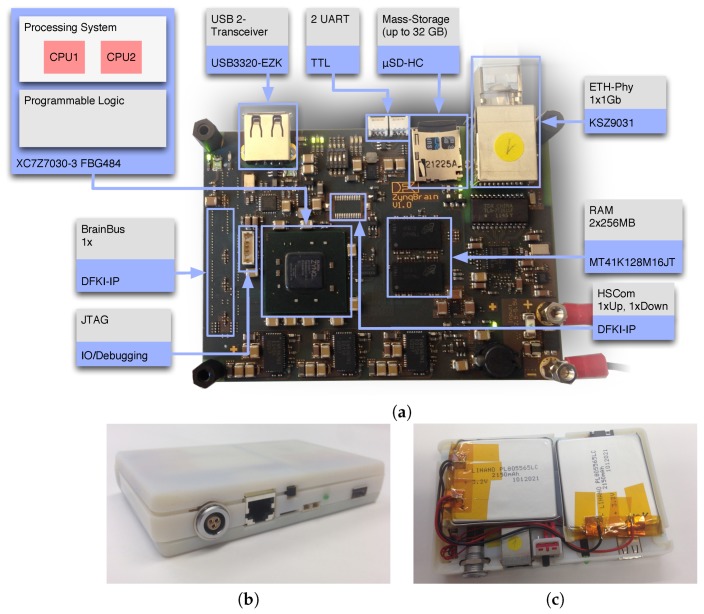
The ZynqBrain processing platform: (**a**) printed circuit board; (**b**) assembled system with 3D-printed cover; (**c**) battery pack.

A schematic diagram of the ZynqBrain is shown in Figure 1a. The ZynqBrain is manufactured in Pico-ITX format (70×100 mm). The central component is a Xilinx Zynq^®^ ZC7030FBG484-3 [40]. A ZC7030 consists of two parts: a Processing System (PS) (which is an ARM^®^ Cortex-A9 dual core CPU running at 1 GHz) and a Programmable Logic (PL) section, which is equivalent to an FPGA. 512 MB DDR3 SD-RAM are connected to the PS.

The amount of programmable resources of the Zynq ZC7030 is shown in Table 1. In all conducted experiments the dual-core ARM Cortex-A9 processor of the ZC7030 was operating at 1 GHz clock frequency, the programmable logic (PL) at 100 MHz.

The ZynqBrain provides the following standardized interfaces to connect to other systems: (1) a single USB 2 interface, (2) a 1 Gb Ethernet port, (3) two UART interfaces and (4) a JTAG port. An important additional feature is the availability of specialized interfaces developed for distributed computing in robotic systems, called BrainBus and HSCom [137]. These interfaces provide the possibility to establish connections to control motors [138] and represent an important key feature for the integration of the ZynqBrain into robotic rehabilitation equipment. The USB interface of the ZynqBrain was used to connect the ZynqBrain directly to the EEG-acquisition hardware (see below), a µSD-HC card interface was used as a persistent storage medium to, e.g., store operating system, software, and recorded EEG data.

For the system used in this paper, we created a 3D-printed cover. Besides the ZynqBrain, the cover contains a power management unit and two LiFePo cells with a total capacity of 4300 mAh.

### 3.2. Dataflow Hardware Accelerator Architecture

**Figure 2:**
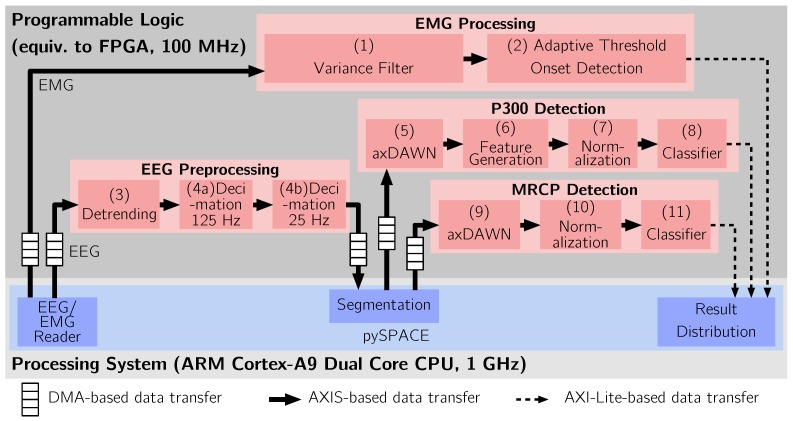
Hybrid parallel processing for electroencephalography (EEG) and electromyography (EMG) detection. The numbers shown in each processing step refer to the detailed description in Section 4.

The processing architecture of the proposed system is shown in Figure 2 (for details about the specific algorithms, see Section 3.3). The PS of the ZC7030 is used for the software parts of the system, while the required Dataflow Hardware Accelerators (DFHWAs) are implemented in the PL.

For each investigated physiological signal, the PL of the Zynq is used to implement a so-called DFHWA. A DFHWA is a task-specific hardware accelerator based on the SDF model (see Section 2.6) and consists of a set of coarse-grain actors. Each actor implements a specific algorithm. Since each physiological signal requires a specific set of operations, the structure of a DFHWA is specific for the corresponding signal. For processing, the data is streamed through the sequence of actors and transformed on its way. Due to the dataflow paradigm, the system is has an inherent parallel architecture that provides the capability to exploit task- and pipeline parallelism to obtain a high computational performance. Task parallelism is especially important for hybrid BCI systems, which have to apply different operations to different streams of data at the same time. Pipeline parallelism represents the separation of a computation in several stages that can be executed in parallel and fits well to stream based computations as required here. Due to the task parallelism, the different DFHWA operate in parallel without interference.

The DFHWAs are implemented using the reSPACE [132,135] framework, which consists of a library of predefined, parametrizable actors. Hence, all actors are configurable components that can be adapted to specific requirements and combined to build more complex systems. These include basic, widely used algorithms such as Finite Impulse Response (FIR) and Infinite Impulse Response (IIR) filters, direct current offset removal, standardization, etc. For instance, for the system proposed in this paper, we use a specific set of actors to build four DFHWAs with different purposes (for details about the applied algorithms see Section 4). Another possibility provided by reSPACE is a customizable generator for matrix-multiply based algorithms, which generates a node according to the specification given by a domain specific language. The operations are mapped directly to the programmable logic to allow resource and power efficient arithmetic operations. This makes it possible to generate nodes that implement algorithms frequently used in BCIs, such as spatial filters [139,140] or classifiers.

The DFHWAs are connected to the CPU via an AXI-Lite bus for the transfer of configuration parameters (for details about the used algorithms, see Section 4) and data. Different parameters of the processing can be configured using a set of software-accessible registers. In order to process EEG or EMG data, it is transferred to the input First-In, First-Out (FIFO) buffers and the results are collected from the output FIFO buffers or result registers. The data transfer is performed using Direct Memory Access (DMA). This setup has the advantage that the host CPU is *not* involved in the data transfer and the computations performed in the DFHWAs and is therefore not occupied by these. Subsequently, the actual computations are performed asynchronously by the DFHWAs. As soon as the results are available, the CPU is notified via an Interrupt ReQuest (IRQ).

To access the DFHWAs from a software application, device drivers are needed that interface with the DFHWAs and run as kernel modules. They can be automatically generated by reSPACE according to the meta-data that provides all necessary information to generate the interface of the DFHWAs.

For the hardware implementation in the corresponding DFHWAs, fixed-point operations were used, while all software implementations are based on double precision floating-point operations. For the proposed system, the integer and fractional parts of the fixed-point operations and representation were manually optimized stepwise to obtain an optimal accuracy.

### 3.3. Software Architecture

The software architecture is based on a customized Linux kernel (version 3.12) with a Debian user space system. This allows us to use the signal processing software framework pySPACE [123] in combination with the DFHWAs. The framework pySPACE is used as the high-level processing and infrastructure software to provide service functionalities, i.e., configuration of the data acquisition and recording, management of previously stored data, and to evaluate and store the obtained results while the actual data processing is performed by the DFHWAs.

The second application of pySPACE in this paper is the usage of pySPACE without hardware acceleration in the reference systems (see Section 5.5). Similar to the structure of the DFHWAs, pySPACE contains a set of signal processing and machine learning algorithms that can be combined to build complex signal processing systems. Note that although pySPACE is written in the Python scripting language, it uses Numpy [141] and Scipy [142] for most operations. Numpy and Scipy contain highly optimized C routines for operations such as matrix algebra and digital filtering. Furthermore, time consuming operations not provided by Numpy or Scipy have been implemented as optimized C extensions and parallelized using OpenMP. Hence, overhead related to the fact that Python is used as a top-level framework language are minimal.

### 3.4. Investigated Physiological Signals

To predict movements, we process the following physiological signals:

- MRCP: Both the ERD/ERS and the MRCP have been used to detect movement intentions (Section 2). Since the MRCPs-based movement-prediction is more reliable than the ERD/ERS-based procedure close to the actual movement onset [143], we choose MRCP for movement prediction in our system.
- EMG: Similarly, the EMG is used as a signal that indicates an upcoming movement.
- P300: The P300 is not directly related to an upcoming movement. However, it can be used to select one of several different modes of a BCI system. In this paper, we follow this approach. It is assumed that the subject is most of the time in an idle state and switches to an active state following the instruction of a human or virtual trainer or therapist. In such an application, the successful detection of the P300 in response to a command can be used as an indicator that the subject will perform a movement in the near future. Subsequent to the command, we expect a movement in a time window with a length of 5 s.

### 3.5. Combination of Signals for Hybrid Movement Prediction

An important point for rehabilitation applications is to obtain a high reliability, i.e., predict upcoming movements as early as possible, but avoid wrong predictions of a movement during rest. In order to achieve this, the individual predictions based on different data modalities can be combined to generate a combined prediction. Clearly, using a combination of several different modalities is computationally more demanding than the usage of a single modality, since it requires to apply different methods to several different data-streams in parallel. The proposed architecture has the advantage that the DFHWAs can operate independently and do not share a common resource, as it is the case when a generic CPU is used. The proposed system provides the possibility to apply all operations described above in parallel to different streams of data, and to collect and merge the predictions of the individual DFHWAs to generate a combined prediction.

Individual predictions can be merged in different ways. The proposed system can either (1) output the prediction of a single DFHWA as system output, or (2) combine the predictions from single DFHWAs and generate a combined prediction. Another possibility would be (3) to merge features of different modalities and use a meta-classifier to generate a common prediction. However, such a solution would be more inflexible, i.e., choosing a different combination would require to retrain the meta-classifier. Hence, approaches (1) and (2) are used in the proposed system.

In particular, we investigate the following single modalities and combinations:

No combination with P300 (NP3):

- MRCP: The prediction of the movement onset is based only on the prediction of the MRCP.
- EMG: The prediction of the movement onset is based only on EMG analysis.
- MRCP or EMG (MoE): The prediction of the movement onset is based on the combination of the MRCP and EMG predictions. The combination is obtained using a *logical or* combination of the single predictions. Formally, let $p_{EMG}\left(t\right),p_{MRCP}\left(t\right)\in \left\{0,1\right\}$ be the predictions of the EMG and EEG of an upcoming movement at time *t*, respectively, where 1 represents the upcoming movement. The combined prediction is then given by $p_{MoE}\left(t\right)=p_{EMG}\left(t\right)\vee p_{MRCP}\left(t\right)$.
- MRCP and EMG (MaE): Similar to MoE, but the combination is obtained using a logical *and*, i.e., $p_{MaE}\left(t\right)=p_{EMG}\left(t\right)\wedge p_{MRCP}\left(t\right)$.

Combination with P300 (CP3):

- P300 and MRCP (PaM): As discussed above, the P300 is used as a switch to select between an *idle* and an *active* state. Specifically, let ${\hat{p}}_{P300}\left(\hat{t}\right)\in \left\{0,1\right\}$ denote the detection of the P300 at time $\hat{t}$. If ${\hat{p}}_{P300}\left(\hat{t}\right)=1$, we set $p_{P300}\left(t\right)\leftarrow 1$ for $t\in [\hat{t}+1\;s,\ldots ,\hat{t}+5\;s]$ and compute $p_{PaM}\left(t\right)=p_{P300}\left(t\right)\wedge p_{MRCP}\left(t\right)$.
- P300 and EMG (PaE): Similar to PaM, but the EMG is used for the movement detection, i.e., we compute $p_{PaE}\left(t\right)=p_{P300}\left(t\right)\wedge p_{EMG}\left(t\right)$.
- P300 and MRCP or EMG (PaMoE): This combination is based on the combinations MoE and PaM, i.e., we compute $p_{PaMoE}\left(t\right)=p_{P300}\left(t\right)\wedge \left(p_{MRCP}\left(t\right)\vee p_{EMG}\left(t\right)\right)$.
- P300 and MRCP and EMG (PaMaE): Similar to PaMaE with $p_{PaMaE}\left(t\right)=p_{P300}\left(t\right)\wedge p_{MRCP}\left(t\right)\wedge p_{EMG}\left(t\right)$.

## 4. Applied Signal Processing and Machine Learning Procedures

In the following, we discuss the applied operations. To compare the performance of the FPGA-based system to CPU-based systems, all operations were implemented two times. The first implementation is based on software components included in our signal processing software framework pySPACE [123]. The software implementation makes use of double precision floating-point arithmetic and represents a reference for comparison. The second implementation is based on the DFHWA architecture discussed in Section 3.2. In this case, all arithmetic operations are implemented using fixed-point arithmetic. The applied operations are shown in Figure 2 and are, apart from the arithmetic type, equivalent for the software and hardware implementation.

### 4.1. EMG Processing

The processing of the EMG was based on the procedure previously presented in [64]. The EMG processing was performed on data segments of 40 ms length, each segment was represented by a 200×c dimensional matrix if the sampling frequency of the EMG recording equipment is 5 kHz, where c∈{1,4,8} refers to the number of EMG channels (see Section 5.2). The processing consisted of two steps:

(1) Variance Filter: To obtain a signal with smoothed baseline noise and enlarged signal amplitudes during movement phases, a running variance method was used for preprocessing of the EMG signals. The calculation was based on [144], but was computed as described in [145] to calculate the running variance *v* at time *t* as:
  (1) $$v\left(t\right)=\frac{1}{W_{VF}-1}\sum_{i=-2w}^{0}x^{2}\left(t+i\right)-{\left(\frac{1}{W_{VF}-1}\sum_{i=-2w}^{0}x\left(t+i\right)\right)}^{2},$$
  where $W_{VF}=2w+1$ is the window length. Variance filtering does not change the dimensionality of the data.
(2) Adaptive Threshold Comparison and Classification: The actual onset detection was based on the comparison of the variance-filtered signal with an adaptive threshold. The threshold was computed as
  (2) $$T\left(t\right)=\mu {\left(t\right)}_{W_{AT}}+p\sigma {\left(t\right)}_{W_{AT}},$$
  with μ the mean value, σ the standard deviation, $W_{AT}$ the length of the window for the mean and standard deviation and *p* the sensitivity factor of the threshold [146]. To compute the standard deviation, the methods described in [145] were used again. The adaptive threshold compensates slow drifts in the EMG signals or changing noise levels in the signal caused by, e.g., resistance changes at the electrode side. The classification was performed using a threshold comparison. That is, a segment was classified as belonging to the movement class if the single sample of the variance filtered signal of more than a previously chosen number of channels $n_{COT}$ were higher than *T*. Due to the adaptive threshold based movement detection, the flow for EMG-based movement prediction required no explicit training phase. A single classification was computed for each data segment.

### 4.2. EEG Preprocessing

The processing of the EEG data for the detection of the MRCPs and P300 was performed in three parts. Each part was implemented by a separate DFHWA. The first part is preprocessing. It was identical for both the P300 and the MRCP. Similar to the EMG processing, the EEG preprocessing is performed on segments of 40 ms length. Each segment was represented by a 200×c dimensional matrix, with c∈{32,64,96,124}. The preprocessing consisted of two operations.

(3) Detrending: First, detrending was used to remove slowly varying signal components using an IIR filter [147], which could otherwise produce a bias in the data.
(4) (a + b) Decimation: Subsequently, the sampling rate was decimated in two steps [148,149], the first step reduced the sampling frequency from 5 kHz to 125 Hz. In the second step, a further reduction of the sampling frequency to 25 Hz was performed. The anti-alias FIR of the second step was parameterized so that all frequencies greater than 4 Hz were attenuated as proposed in [150,151]. In the decimation, each data segment is reduced to a *c* dimensional vector. The output of the second decimation step was sent back to the main memory for further segmentation in software.

### 4.3. Data Segmentation

Before the data was processed by further processing methods, the preprocessed segments were combined to segments segments specific for the P300 or MRCP processing. The segments were transferred to the corresponding DFHWA and processed independently from each other.

### 4.4. P300 Processing

For the P300, segments of 1000 ms in length were created by assembling 25 preprocessed segments, whenever a target or standard marker was shown to the subject. Hence, each segment was represented by a 25×c dimensional matrix. For training and evaluation, we used a dataset-wise cross validation as discussed in Section 5.3. The P300 processing consisted of four steps.

(5) Spatial Filtering: The axDAWN spatial filter [140] was applied to decrease the number of channels to four, creating a 25×4 dimensional matrix. This operation can be realized as a matrix multiplication (using DSP48 [121] slices if realized in hardware) and was implemented using a specialized accelerator for matrix operations [152].
(6) Feature Generation: To generate features for classification the time samples were transformed into local straight line features, i.e., polynomial features of order one [47,57]. To this end, every segment was divided into subsegments of length of 400 ms that are shifted by 120 ms. A polynomial was fitted to each subsegment. The polynomials allow to describe the P300 by a series of slope values. The extracted slopes were combined in a single 24-dimensional feature vector.
(7) Feature Standardization: A further operation was component-wise feature standardization, i.e., we computed $x_{s}\leftarrow \frac{x-\mu_{T}}{\sigma_{T}}$, where $\mu_{T},\sigma_{T}$ are the element-wise mean and standard deviation of all vectors in the training data set, respectively. This operation removes bias from the data due to a constant offset. Feature standardization does not change the dimensionality of the feature vector.
(8) Classification: A Passive-Aggressive Algorithm, type-I (PA-1) [153] was used for classification. The PA-1 classifier is a linear binary online classifier, that is based on a maximum-margin hyperplane and can be applied to nonseparable problems. Preliminary investigations showed no significant differences in classification performance to a linear soft-margin Support Vector Machine (SVM). Similarly to a soft-margin SVM, the PA-1 provides a hyperparameter *C* to control regularization. *C* was optimized using a grid-search in the range $\left\{10^{0},10^{-1},\ldots ,10^{-6}\right\}$. A stratified three-fold nested cross validation was used for model selection on the training data [154]. Subsequent to the classification, a threshold correction [155] was used to compensate the bias in the data related to class imbalance.

### 4.5. MRCP Processing

**Figure 3:**
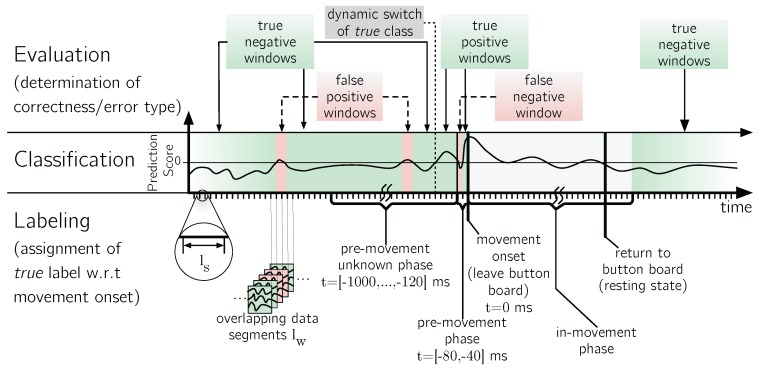
Segment-based evaluation. The true label was assigned to each segment based on the temporal relationship to a movement onset. Since we observed highly subject-specific differences of the time when a movement can be predicted before the actual movement onset, we used the following dynamic method to determine whether the true class of a segment is the movement or rest. In all cases the segments ending at −80 or −40 ms relative to the movement onset were expected to predict a movement (i.e., belong to the movement class, denoted as pre-movement phase). All segments ending in the interval [−1 s,…,−120 ms] were further investigated whether they belong to a consecutive sequence of predictions of the movement class with at most one exceptional case (pre-movement unknown phase). All segments ending between the movement onset and the end of the movement plus an additional tolerance period of 200 ms were not included in the performance evaluation (post-movement phase). All remaining segments were assigned to the resting class. For training of the classifier, the segments ending in the interval [−120,…,120] ms were used as examples for the movement class, all remaining segments (apart from examples ending in the in-movement phase) were used as examples for the resting class.

Since we are interested in the exact timing of the EEG-based movement prediction, we use MRCP-based procedures for the proposed system. MRCPs can be represented in the time-domain. For the MRCP detection, overlapping segments (see Figure 3 in Section 5.4), with a duration of 200 ms were used. Similarly to the EMG, a prediction was performed every 40 ms, adjacent segments overlapped by 160 ms. Each segment consisted of c×5 samples, where *c* is the number of channels. For training of the spatial filter, classifier and feature standardization, the segments of the training phase were labeled as related to a *movement* or to a *rest* phase based on the temporal relationship to the movement onsets as shown in Figure 3. Similar to the P300 processing, we used a dataset-wise cross validation as discussed in Section 5.3.

(9) Spatial Filtering and Feature Generation: Since the MRCP is reflected by a temporal pattern in the data, time-domain features can be used for the detection of the MRCP. Accordingly, the axDAWN spatial filter [140] can be applied for the MRCP detection to reduce the number of channels to a single channel, i.e., creating a 20 dimensional feature vector.
(10) Feature Standardization: The feature vectors were standardized using the approach discussed in Section 4.4 (7), leaving the dimension of the feature vector unchanged.
(11) Classification: The 20 dimensional feature vector was classified using a Passive-Aggressive Algorithm, type-I [153]. Similar to the P300 detection, the regularization hyper-parameter *C* was optimized in the range $\left\{10^{0},10^{-1},\ldots ,10^{-6}\right\}$ using a 3-fold nested cross validation. Again, we observed no significant differences in classification performance to a linear soft-margin SVM in preliminary investigations and applied a threshold correction to compensate class imbalance as proposed in [155].

## 5. Experimental Evaluation

The evaluation was split into two different parts:

1. A preliminary offline evaluation to test and evaluate the system regarding different parameters and combination of modalities and select the best parameters for the online evaluation.
2. A subsequent online evaluation to verify that the system works in a real application.

The experimental setup was identical for both the online as well as the offline part.

### 5.1. Experimental Setup

In order to evaluate the proposed system, we developed an experimental setup that combines an oddball paradigm with movement prediction.

Twelve healthy male subjects with normal or corrected to normal vision participated in this study, eight subjects participated in the offline part (age 29.9±3.3 years) and four subjects in the online part (age 26.8±1.3 years). All participants gave written consent for participation and the study was approved by the ethics committee of the University of Bremen. In order to obtain a sample size sufficient for quantitative analysis, the current experiment was conducted by healthy subjects.

**Figure 4:**
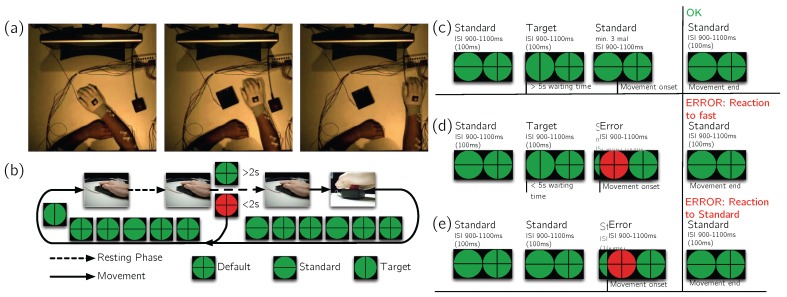
Illustration of the conducted experiments. (**a**) Three pictures of the setup, showing from left to right: the resting phase, the movement phase and the buzzer press; (**b**) Visualization of the paradigm; (**c**) Correctly performed movement; (**d**,**e**) Notification of incorrectly performed movements.

Figure 4 shows the experimental setup and procedures. The subjects were seated in a chair in front of a table. A monitor, a button board and a buzzer were placed on the table (Figure 4a). The subjects were asked to perform 40 self-paced movements of their right arm according to an oddball paradigm. In the resting state, the hand of the subject was placed on the board. To perform a correct movement, the hand of the subject had to leave the button board, press the buzzer, and return to the button board. The distance between buzzer and button board was approximately 30 cm. Events from button board and buzzer were labeled in the acquired data to determine the movement onset and the end of the movement.

**Figure 5:**
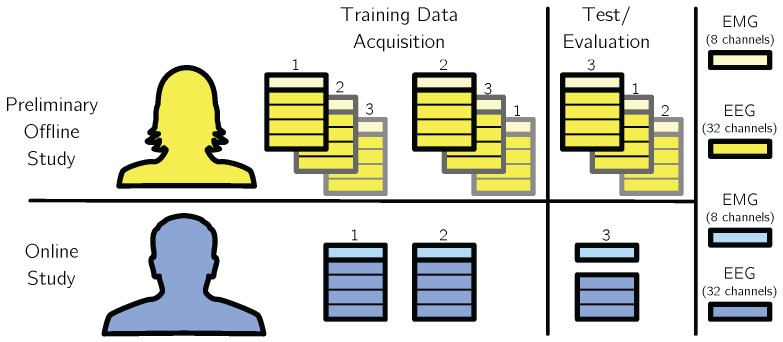
Illustration of the online evaluation/offline cross validation scheme (see also Section 5.3). In the preliminary offline study, three runs of data were recorded in total. The runs of the data were used as splits for the cross validation as follows. All combinations of runs in a session were used, resulting in three different training/evaluation combinations. Two recorded runs were used for training of the data dependent methods and the remaining run was used for the offline evaluation. In each run the data was recorded using the Magma-Box-Setup, i.e., it consisted of 4×32=128 channels of EEG data and 8 channels of EMG data. Due to the restrictions of the recording hardware discussed in the text, a modified recording scheme was used for the offline evaluation. Online evaluation: In this case, the two initial training sets were recorded using the Magma-Box-Setup. In the test/evaluation phase, a single run was recorded using the USB-Box-Setup. The physiological data was processed in real-time and the results recorded for subsequent analysis.

The oddball paradigm was implemented using the software Presentation (Neurobehavioral Systems, Inc., Albany, CA, USA). A green circle with a black fixation cross was shown to the subjects. Two different kinds of stimuli were shown to the subjects: Standards, which were indicated by the disappearance of the vertical bar of the fixation cross, and Targets, which were indicated by the disappearance of the horizontal bar for 100 ms. The Inter-Stimulus Interval (ISI) was 1 s ±100 ms (Figure 4b). After a Target stimulus, the subjects had to wait for at least 2 s before they were allowed to perform a valid movement (Figure 4c). The waiting time was unknown to the subjects. Movements performed too early (Figure 4d) or not related to a Target stimulus (Figure 4e) were reported to the subjects by a red flash of the circle as error notification and not taken into account for data analysis. A run was finished after 40 correct movements. The study consisted of one recording session per subject, each recording session was divided into three runs as shown in Figure 5 (resulting in 120 movements per subject and session; 80 movements belong to the training and 40 movements to the test phase). 24 datasets from 8 subjects (3runs×8subjects) were used in the offline evaluation, and 4 datasets from 4 subjects (1runs×4subjects) for the online evaluation.

### 5.2. Data Acquisition

EEG and EMG data were recorded at 5000 Hz with a 128 electrode (extended 10–20 system) actiCAP system using four 32 channel BrainAmp DC amplifiers (BrainProducts GmbH, Munich, Germany) for the EEG and a BrainAmp ExG MR bipolar EMG-System for the 8 EMG channels. EMG was acquired using bipolar Ag/AgCl gel electrodes at four muscles of the right arm: M. brachioradialis (Channel 1), M. bizeps brachii (Channel 2), M. triceps brachii (Channel 3), and M. deltoideus (Channel 4). For comparison reasons, a similar electrode arrangement was attached to the left arm for Channels 5–8. Due to restrictions of the recording hardware, two different setups were used for data acquisition:

- Magma-Box Setup: In this setup, a Magma box with two Brain Products amplifier PCI cards were used, the first PCI card was used to acquire 128 channels of EEG, the second card to acquire 8 channels of EMG data. Electrodes I1, OI1h, OI2h, and I2 of the EEG were used for recording the electrooculogram, which is not considered in the following analysis. The Magma-Box setup was used for the offline evaluation and recording of the training data for the online evaluation.
- USB-Box Setup: In this setup, a Brain Products USB 2 Adapter was used to record the data. Since it is restricted to four BrainAmp DC amplifiers, three amplifiers were used for EEG data acquisition and one amplifier was used for EMG data acquisition.

To connect the actiCAP system in the USB-Box Setup to the ZynqBrain, the device drivers provided by the vendor have been ported to Linux. The maximal number of EEG and EMG channels used in the evaluation depends on the setup (see Figure 5 for details). During acquisition, the data was filtered initially by the recording hardware with a passband between 0.1 and 1000 Hz.

### 5.3. Evaluation Procedures

The evaluation scheme is shown in Figure 5. For the offline analysis, a 3-fold cross-validation was used in all evaluations. Two of the three recorded runs were used for training of the machine-learning and data-dependent signal processing methods. The remaining run was used for offline evaluation. This was performed for all possible combinations of training and evaluation runs. By using the predefined splits of the data as given by the runs, the temporal order inside each split is retained in order to apply the segment-based evaluation as shown in Section 5.4. In the online evaluation, only the first two runs were used for training, while the third run was used for online evaluation. No testing data was used in the training phase and vice versa.

For the offline evaluation, 124 EEG channels were recorded; in the offline analysis, we used a fixed number of 96 channels due to recording hardware restrictions discussed above.

The processing was performed in order to detect and combine three different potentials in EEG or EMG data, as shown in Figure 2. To achieve this, it consists of four DFHWAs with specific tasks, as described below. In all cases, the data acquisition was performed using pySPACE, which is capable to read data from files (for offline analysis) or acquire and process data directly from EEG systems (for online analysis). To evaluate the DFHWAs, the data was then transferred to the DFHWAs implemented in the PL via DMA. Furthermore, all acquired data was recorded in files for subsequent analysis.

Three key metrics were of interest for the study provided in this manuscript: classification performance, computing performance, and power consumption.

### 5.4. Classification Performance

We analyze the classification performance for the following purposes. First, we want to determine the number and combination of required channels that give the best results. The processing time of all systems and the required PL resources of the DFHWAs depends on the number of channels. Hence, a small number of channels can result in a low processing time, but can also affect the classification performance. Second, we want to compare the different modalities and combinations regarding their classification performance. Third, as discussed above, the CPU implementations were using double precision floating-point arithmetic, while the FPGA-based system was using fixed-point arithmetic. Since fixed-point arithmetic can result in numerical problems, a further concern was that this might decrease the classification accuracy.

We used a segment-based method for performance evaluation for MRCP and EMG (see Figure 3), since this provides the possibility to evaluate the system under conditions close to a real application case, where a movement prediction system should perform an ongoing analysis of the physiological data. For the P300 detection, we use segments of the length of 1 s subsequent to each Standard and Target marker.

Regarding the classification performance, we are interested in three performance measures: the overall classification performance in terms of Balanced Accuracy (BA), as well as the types of errors as given by the False Negative Rate (FNR) and False Positive Rate (FPR). The BA is given by the arithmetic mean of the True Positive Rate (TPR)/recall and True Negative Rate (TNR)/specificity, i.e.,

$$BA=\frac{1}{2}TPR+\frac{1}{2}TNR,$$

with $TPR=\frac{TP}{TP+FN}$ and $TNR=\frac{TN}{TN+FP}$, where *TP, TN, FP, FN* denote the true positives, true negatives, false positives and false negatives, respectively. The BA was selected for the performance assessment, as it allows a simple interpretation when the class frequencies are highly imbalanced [156] as it is the case here, where the segment-based evaluation results in a class-ratio of ≈1:110 for movement vs. rest. Correspondingly, the FPR and FNR are given by $FNR=\frac{FN}{TP+FN}$ and $FPR=\frac{FP}{TN+FP}$.

### 5.5. Computing Performance: Reference Systems for Comparison

The computing performance of the proposed system (denoted as FPGA-based system) was compared to the following CPU-based reference systems:

- A mobile processor-based system (the dual core ARM CPU of the ZC7030 PS, running at 1 GHz, 512 MB DDR SDRAM at 533 MHz), denoted as Mobile CPU-based Reference System (MRS) in the following.
- A standard desktop PC with an 8-core Intel(R) Core(TM) i7 CPU that was running at 3.07 GHz and a Linux Ubuntu operating system, version 14.4. denoted as Standard-PC Reference System (SRS) in the following.

In preliminary performance investigations, we identified the application of the anti-alias FIR filter applied as part of the decimation operation (see Section 4) as the computationally most expensive step. Hence, we used OpenMP [157] to apply a channel-wise parallelization of the filtering procedure.

All unnecessary processes were stopped to avoid a distortion of the results. As stated above, we used pySPACE [123] for processing and evaluation of the software parts of all systems.

### 5.6. Measurement of Power Consumption

The power consumption is an important aspect for processing systems, especially if the systems should be used inside a rehabilitation device independent from a stationary power supply. The total input power consumption of all systems was measured using a Tektronix TCP312/TCPA300 current clamp/amplifier.

## 6. Results and Discussion

The results are divided into two parts. The first part (Section 6.1, Section 6.2, Section 6.3, Section 6.4, Section 6.5 and Section 6.6) contains the results of the offline evaluation which was conducted to determine the optimal system configuration and parameters for the subsequent online evaluation session. Furthermore, the offline evaluation permits us to evaluate the classification accuracy of the single signals as well as their combinations. The second part (Section 6.7) reports the results of the validation of the developed system in the subsequently performed online sessions.

### 6.1. EMG-Based Movement Prediction: Classification Performance, Computing Time, Resource Utilization

*Classification Performance:* A grid search was used for optimization of $W_{VF}$, $W_{AT}$, and *p*. Based on previous experience, the chosen parameter ranges for optimization were $W_{VF}\in \left\{10,20,50,100,200\right\}$ ms, $W_{AT}\in \left\{100,200,500,1000,2000,5000\right\}$ ms, p∈{3,…,12}. Regarding the channel setup, we estimate the performance of each single channel as a baseline for comparison, and the following combinations of channels: $n_{COT}\in \left\{1,2,4\right\}$ for channels 1,…,8 and $n_{COT}\in \left\{1,2\right\}$ for channels 1,…,4, where $n_{COT}$ denotes the number of channels that have to be over the computed threshold simultaneously (see Section 4.1).

**Figure 6:**
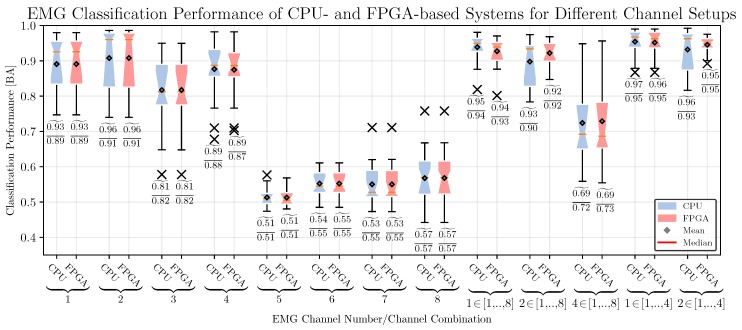
Classification performance for the prediction of movements based on the EMG. The median ($\tilde{BA}$) and mean ($\bar{BA}$) classification performance is reported for each case.

To this end, the classification performances of eight single-channels and five different channel combinations (see Figure 6) were analyzed by a two-way repeated measures ANOVA with the channel setup (CS, 13 levels as described above) and hardware architecture (HA, 2 levels: CPU-based system with double precision floating point vs. FPGA-based system with fixed-point arithmetic) as within-subjects factors. Greenhouse-Geisser correction was applied when necessary and the corrected *p*-value is reported. We performed a post-hoc analysis to compare the channel setups and hardware architecture. Bonferroni-Holm correction was applied for multiple comparisons.

We obtained a significant main effect for CS [F(12,276)=211.5, p<0.001], but no effect of HA [F(1,23)=2.2, p=n.s.]. The best channel setups are ‘1∈[1,…,4]’ and ‘1∈[1,…,8]’ [these setups vs. all other setups: p<0.01]. There was no significant difference between CPU and FPGA.

Furthermore, we observe a significant interaction between CS and HA [F(12,276)=6.2, p<0.001]. We found no significant differences between both types of hardware architectures for all channel setups [p=n.s.], except for channel 1, and for all combinations except ‘2∈[1,…,4]’.

As expected, the channel placed on the left arm show worse performance compared to the other channel setups [p<0.001]. Combinations of multiple channels improve the performance, the best performance is obtained by channel setups ‘1∈[1,…,8]’, ‘2∈[1,…,8]’ and ‘4∈[1,…,4]’. Based on these results, all subsequent investigations related to the EMG based movement prediction are based on the setup ‘1∈[1,…,4]’.

*Computing Time:* Computing times were measured using all 40 ms segments from datasets of the offline study, in total this data consists of N= 381,991 segments. All measured times contain the time spent in the core algorithmic kernel and the time required to make the results accessible to the Python-based top-level framework pySPACE, e.g., for the FPGA-based system this includes the time required to perform the data transfer between main memory and hardware accelerator.

**Figure 7:**
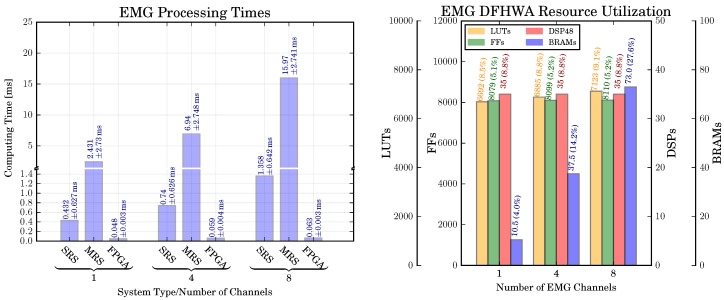
Left: Computation time for different computing setups for the detection of the movements based on the EMG. Right: Resource utilization of the Dataflow Hardware Accelerator (DFHWA) for different numbers of EMG channels. The percentage values refer to the amount of resources provided by the Zynq ZC7030 (see Section 3.1) (independently utilized 18 kb Block Random Access Memorys (BRAMs) are reported as 0.5 BRAMs).

The required time to process a segment of the proposed system (FPGA) and the reference systems (MRS and SRS, see Section 5.5) is shown in Figure 7 (left part). We can observe that the required time to process a segment depends on the number of channels and the utilized computing device. For all channel setups, the MRS requires the highest amount of time (2.431–15.97 ms, depending on the number of channels) and has the highest variability of time ($\sigma_{t}\approx 2.7$ ms). The SRS provides a significant speedup over the MRS system (5.6–11.8×, depending on the number of channels) and has a smaller variability of the processing time ($\sigma_{t}\approx 0.6$ ms). The highest performance and lowest variability of processing time can be obtained by the FPGA-based system (speedup over SRS: 9.0–21.6×, speedup over MRS: 50.6–253.5×, $\sigma_{t}\approx 0.003$ ms). The required time to process a segment represents a delay that might be, depending on the actual application and the related time constraints, unacceptable.

*Resource Utilization:* The resource utilization is shown in Figure 7 (right part). It can be observed that there is only a small increase in the amount of utilized LUTs, FFs, and DSP48 for an increasing number of channels. In contrast to this, the increase of utilized BRAMs is more pronounced (10.5 BRAMs are required for a single channel, 37.5 BRAMs for four channels, and 73 BRAMs for eight channels). The reason for this increase of utilized BRAMs is the requirement to increase the buffer sizes of the variance filter and adaptive threshold computations to compute the sliding mean and variance (see Section 4.1), whereas the actual computational units are used in a multiplexed fashion for different data samples and channels.

### 6.2. MRCP-Based Movement Prediction: Classification Performance, Computing Time, Resource Utilization

*Classification Performance:* The classification performance for different numbers of channels according to the extended 10–20 system and both hardware architectures was analyzed using a repeated measures ANOVA with two within-subjects factors: numbers of channels (NC, 4 levels: $n_{c}$ channels with $n_{c}\in \left\{32,64,96,124\right\}$) and type of hardware architecture (HA, 2 levels: {CPU,FPGA}). Greenhouse-Geisser correction was applied when necessary. Bonferroni-Holm correction was applied for multiple comparisons.

**Figure 8:**
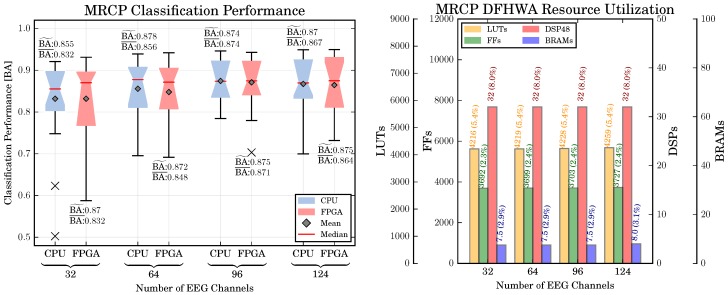
Left: Classification performance for the detection of the MRCP for CPU and FPGA for 32–124 channels. The median ($\tilde{BA}$) and mean ($\bar{BA}$) classification performance is reported for each case. Right: Resource utilization of the DFHWA for different numbers of EEG channels. The percentage values refer to the amount of resources provided by the Zynq ZC7030.

The obtained classification performance is shown in Figure 8. A significant main effect can be observed for NC [F(2.07,47.6)=12.7, p<0.01], but not for HA [F(1,23)=1.18, p=n.s.]. The effect of number of channels is not straightforward for each architecture type. In our application, the highest BA is achieved with 96 channels (except for 124 channels) [$n_{c}=96$ vs. $n_{c}=32$: p<0.001, $n_{c}=96$ vs. $n_{c}=64$: p<0.001, $n_{c}=96$ vs. $n_{c}=124$: p=n.s.].

**Figure 9:**
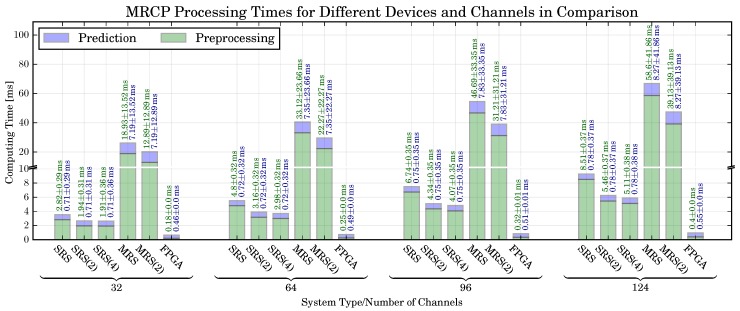
Computing times of different computing setups for 32–124 channels for the detection of the Movement Related Cortical Potential (MRCP). For the Standard-PC Reference System (SRS) system, the number of threads are put in parenthesis.

*Computing Times:* The computing times for the different reference systems discussed in Section 5.5 are shown in Figure 9. Reported is the amount of time required to perform a single prediction, i.e., to process 200 ms of EEG data. Two values are reported for each setup: the time required for the preprocessing (which corresponds to the Preprocessing DFHWA shown in Section 4.2) as well as the time for the actual prediction (which corresponds to the MRCP Processing DFHWA). It can be observed that, independent from the number of channels, the processing times of the SRS are sufficiently fast to predict movements online, i.e., they require less than 40 ms of time to process a segment of 40 ms of EEG data. We can only observe a small speedup by parallelizing the preprocessing (the smallest speedup of 47% is obtained for 32 channels, the highest speedup of 66% can be obtained for 124 channels). The reason for this observation is a high proportion of serial code related to object construction and data exchange between nodes in pySPACE. A separate investigation (not shown) revealed that the speedup increases if the size of the data segments is increased. However, this would also result in an increase of the latency related to each prediction. Hence, a larger segment size is not feasible for the application of online movement prediction.

The MRS requires a significantly higher amount of time for each prediction. For a small number of channels ($n_{c}\in \left\{32,64\right\}$), it is possible to use the MRS for online prediction (i.e., less than 40 ms are required to process a segment). However, for these channel setups we obtain a classification performance worse than for $n_{c}\in \left\{96,124\right\}$. Hence, an optimal system configuration would consist of a configuration with a higher number of channels, i.e., $n_{c}\in \left\{96,124\right\}$. For such a configuration the MRS does not provide a sufficient amount of computing performance for an online application.

If the DFHWAs are used, the computation times can be considerably reduced and the time constraints for real-time prediction are met. The achievable speedup depends on the number of channels. For 32 channels, achievable speedup is the lowest (FPGA vs. SRS: ≈4–5.5, FPGA vs. MRS ≈31.4–40.8), the highest speedups can be achieved for 124 channels (FPGA vs. SRS: ≈9.8–6.2, FPGA vs. MRS: ≈49.9–70.4).

*Resource Utilization:* A further concern was again the amount of required resources in relation to the number of channels. The resource utilization for different PL resources and number of EEG channels is shown in Figure 8 (right). It can be observed that the increase of resources for an increasing number of EEG channels is generally low, the highest increase of resources can be observed for the required LUTs in the preprocessing part of the MRCP (32 vs. 124 channels: 249.7%). A smaller increase can be observed for the FFs (32 vs. 124 channels: 10.3%), while no increase can be observed for the BRAMs and DSP48 slices. The reason for this observation is attributable to the *delay* scaling technique applied to scale the system to different numbers of channels. As discussed in Section 3.2, the application of delay scaling increases the number of registers in a DFHWA (which are implemented as LUTs and FFs), but not the number of multipliers and adders (which are mapped to DSP48 slices).

### 6.3. P300 Detection: Classification Performance, Computing Time, Resource Utilization

**Figure 10:**
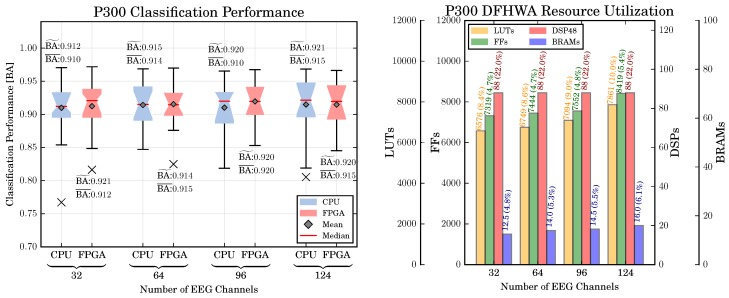
Left: Classification performance of the P300 detection for CPU and FPGA for 32–124 channels. Right: Resource utilization of the DFHWA for different numbers of EEG channels. The percentage values refer to the amount of resources provided by the Zynq ZC7030.

*Classification Performance:* The classification performance for the CPU- and FPGA-based systems of the P300 is shown in Figure 10 (left part). In addition, the The mean and median classification performance over all numbers of channels and both hardware architectures are 0.91±0.04 and 0.92, respectively. Hence, in comparison to the MRCP, the P300 can be detected more reliable. For the P300 detection, we also investigate the effect of the number of channels and hardware architecture on the classification performance. To this end, we perform a two-way repeated measures ANOVA with the numbers of channels $n_{c}\in \left\{32,64,96,124\right\}$ and the hardware architecture HA={CPU,FPGA} as within-subjects factors. Similar to the MRCP evaluation, we obtain no difference of the classification performance between both hardware architectures [F(1,23)=1.4,p=n.s.]. However, in contrast to the MRCP evaluation we obtain also no difference in the classification performance between the numbers of channels [F(3,69)=1.5,p=n.s.].

**Figure 11:**
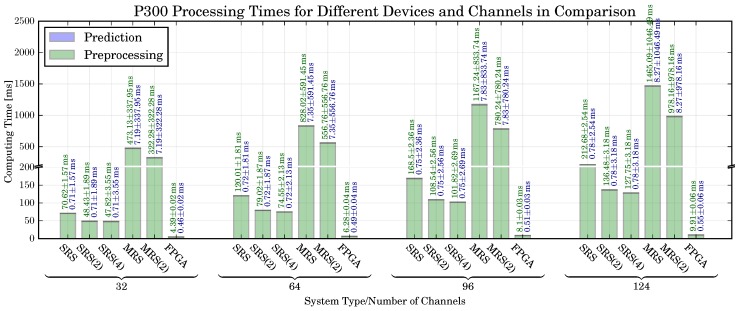
Computing times of different computing setups for 32–124 channels for the detection of the P300.

*Computing Time:* The P300 processing times are shown in Figure 11. The computing time refers to the time required to classify a single segment of EEG data. As discussed in Section 4.4, a corresponding segment has the length of 1000 ms. It can be observed that, similar to MRCP processing times, the time depends on the number of channels and the utilized system. The highest amount of time is required by the MRS. However, in this case the MRS is capable to perform online processing if less than 124 channels are used. Similar to the MRCP and EMG processing, the FPGA-based solution provides a significant speedup over the MRS and SRS.

*Resource Utilization:* The resource utilization for different PL resources is shown in Figure 8 (right). Similar to the MRCP DFHWA resource utilization, it can be observed that the increase of resources for an increasing number of channels is generally low. However, due to higher number of processing steps, the resource utilization is higher than the MRCP DFHWA resource utilization.

### 6.4. Hybrid Movement Prediction: Classification Performance, Prediction Time

The last step in the offline analysis was to determine if the combination of different modalities as described in Section 3.5 can improve the classification performance. The classification performance shown in Figure 6 and Figure 8 reveals that the EMG-based movement prediction provides a higher classification performance than the MRCP-based movement prediction. However, it is well known that the EEG allows a prediction of movements earlier than the EMG. Hence, an important point for the usage in a rehabilitation device is to study the effect combining multiple predictions on the overall classification performance, reliability and prediction time. The proposed system provides the possibility to process several data streams in parallel. Hence, it permits to apply the hybrid combinations described in Section 3.5.

**Figure 12:**
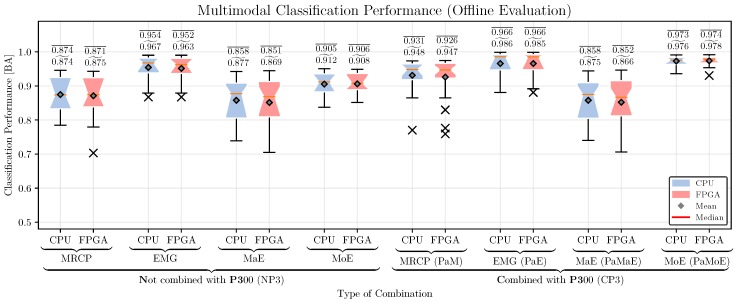
Classification Performance for Hybrid Movement Prediction.

*Classification Performance:* The classification performance of the hybrid combinations for the CPU- and FPGA-based systems is shown in Figure 12. For comparison, we performed a three-way repeated measures ANOVA with the movement prediction method (method, 4 levels: EMG, MRCP, PaM, PaE), combination with P3 (P3, two levels: NP3, CP3), and hardware architecture (HA, 2 levels: CPU, FPGA) as within-subjects factors. Greenhouse-Geisser correction was applied when necessary and the corrected *p*-value is reported. For post-hoc comparisons, we performed paired t-tests. Bonferroni-Holm was applied for multiple comparisons. We obtained significant main effects for method [F(3,69)=43.6, p<0.001] and P3 [F(1,23)=222.4, p<0.001], but no effect of HA [F(1,23)=1.5, p=n.s.].

The best method is EMG, followed by MoE, MRCP, and MaE [p<0.001 for all pairs of methods]. A combination with the P3 improves the classification performance [CP3 vs. NP3: p<0.001]. There was no difference in classification performance between CPU and FPGA [p=n.s.].

Furthermore, we observe a significant interaction between movement prediction method and P3 combination [F(3,69)=80.6, p<0.001]. The best performance is obtained by EMG, followed by MoE, irrespective if it is combined with P3 or not [p<0.05]. However, for the MRCP we observe an increased performance compared to MaE only when it is combined with the P3 [MRCP vs MaE: *p* < 0.05 for P3, p=n.s. for NP3]. P3 improves the classification performance for all methods except for MaE [p=n.s. for MaE, p<0.05 for other methods]. The hardware architecture interacts with neither P3 nor methods.

Finally, there was no interaction between HA, P3 and methods [F(3,69)=1.3,p=n.s.]. The post-hoc analysis reveals that the combination with the P3 improves the classification performance for all methods [p<0.001], except for MaE [p=n.s.]. This pattern is observed for both architectures. Furthermore, for both CP3 and NP3 there was no difference in classification performance between both architectures for all methods [p=n.s.]. A combination with the P300 improves the classification performance for all methods except for MaE [p=n.s. for MaE, p<0.01 for other methods]. This pattern was observed again for both architectures.

Another important point in the direct comparison of different combinations is a consideration of the errors rates and precision. The mean FNRs, FPRs and recall for the combinations and both hardware architectures is given in Table 2. For comparison, we performed three-way ANOVAs with movement detection method, combination with P300, and hardware architecture as within-subjects factors, which were performed separately for the FNR, FPR and precision as dependent variables, respectively.

For the FNR, we observe a significant main effect for the movement prediction method [F(3,69)=72.8, p<0.001], but neither for combination with P300 [F(1,23)=2.7, p=n.s.] nor hardware architecture [F(1,23)=0.8, p=n.s.]. The lowest FNR is obtained by MoE, followed by EMG, and MRCP; the highest FNR is obtained for MaE [p<0.001 for all pairs of methods].

For the FPR, we observe significant main effects for movement prediction method [F(3,69)=131.2, p<0.001] and combination with P300 [F(1,23)=240.5, p<0.001], but not for HA [F(1,23)=0.2, p=n.s.]. Regarding the FPR, the best method is EMG, followed by MoE, MRCP, and MaE [p<0.001 for all pairs of methods]. A combination with the P3 improves the FPR [CP3 vs. NP3: p<0.001]. There was no difference in FPR between CPU and FPGA [p=n.s.].

For the FPR, we observe again a significant interaction between movement prediction method and P3 combination [F(3,69)=120.8, p<0.001]. A further analysis reveals that the combination with the P3 reduces the FPR for all methods [p<0.001]. This pattern is observed for both architectures. Furthermore, for both NP3 there was a difference in FPR between both architectures for all methods [p=n.s.]. A combination with the P300 improves the FPR for all methods [p<0.01]. This pattern was observed again for both architectures.

The precision depends substantially on the applied signal combination method. For the precision, we observe significant main effects for movement prediction method [F(3,69)=205.5, p<0.001], combination with P300 [F(1,23)=797.4, p<0.001], and for HA [F(1,23)=4, p<0.05]. Regarding the precision, the best method is MaE, followed by EMG, MRCP, and MoE [p<0.001 for all pairs of methods except MRCP vs. MoE: p=n.s.]. A combination with the P3 improves the precision [CP3 vs. NP3: p<0.001]. For the precision, we observed a difference between CPU and FPGA [p<0.01].

Furthermore, we observe again a significant interaction between movement prediction method and P3 combination [F(3,69)=12.0, p<0.001] and additionally a significant interaction between method and HA [F(3,69)=6.5, p<0.001]. A further analysis reveals that the combination with the P3 improves the precision for all methods [p<0.001]. This pattern is observed for both architectures. Moreover, for NP3 there was a difference in precision between both architectures for EMG and MaE [p<0.01], but not for MRCP and MoE [p=n.s.]. For CP3, there was no difference in precision for both architectures for all methods [p=n.s.]. A combination with the P300 improves the precision for all methods [p<0.01]. This pattern was observed again for both architectures.

**Figure 13:**
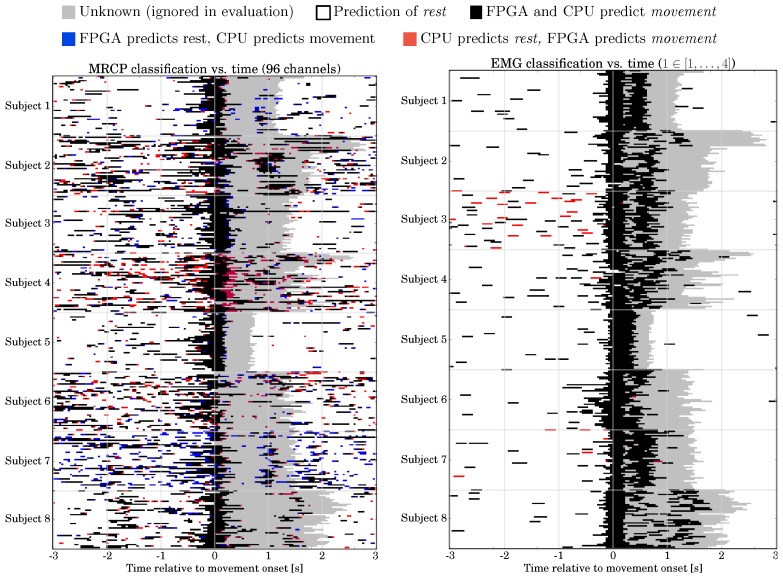
Predictions relative to the movement onset of the CPU and FPGA-based systems for the MRCP- and EMG-based movement prediction. The predictions of movements for the interval [−3 s, 3 s] is depicted relative to each movement onset for MRCP-based predictions (left) and EMG-based predictions (right). Predictions of rest are left white, the unknown class subsequent to each movement is shown with a grey background. The prediction of a movement is encoded as follows: the prediction of a movement by the CPU-based systems as well as the FPGA-based system is shown in black. The prediction of a movement by the CPU-based system but not the FPGA -based system is shown in blue, the opposite case is shown in red.

*Summary:* We observe that the MRCP-based movement prediction is less accurate than the EMG-based prediction. This can also be observed for the individual trials shown in Figure 13. The reason for the lower accuracy might be the detection of a movement planning or intention that does not result in a movement execution [10,158,159]. The classification performance is improved by the combination of the MRCP with the P300 and/or EMG. Depending on the type of combination, different properties regarding FPR, FNR and precision can be observed. The combination of the MRCP with the EMG by a logical and decreases the FPR, but increases the FNR. The opposite can be observed for the FNR and precision. In an additional combination with the P3000, we observe a decrease of the FPR, but an increase of the FNR for the MRCP and MoE. While the decrease of the FNR is only small for the MRCP, we can observe a significantly higher increase for MaE. In this case, up to 25% of the movements are missed. A decrease of the FPR can be observed for the MoE combination. The precision is increased in general by a combination with the P300.

### 6.5. Prediction Time

**Figure 14:**
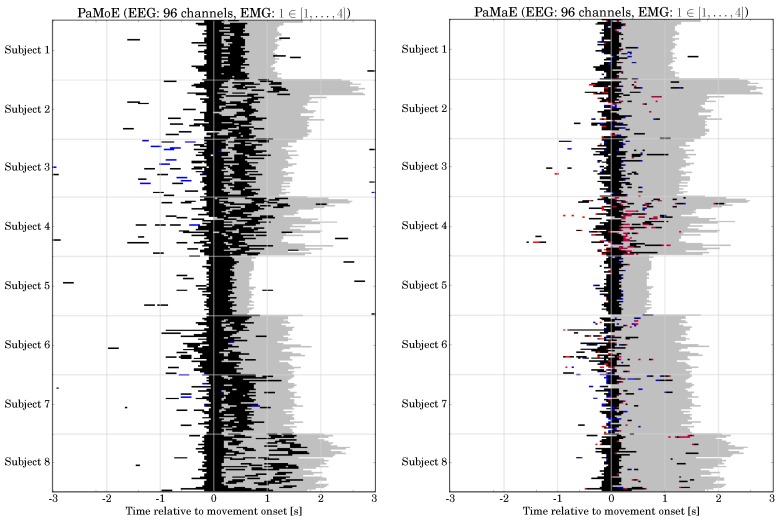
Predictions relative to the movement onset of the CPU and FPGA-based systems for the PaMaE and PaMoE combinations. For a more detailed figure description, see Figure 13.

An important point in the comparison of different modalities is not only the classification accuracy, but also the capability to predict an upcoming movement as early as possible. We define according to [25] the prediction time as the earliest point in time in the pre-movement unknown phase that predicts a movement onset. The mean ($\mu_{t}$) and 75/50/25% quartile ($Q_{t}^{75},Q_{t}^{50},Q_{t}^{25}$) prediction times for the EEG, EMG and all investigated combinations are shown in Table 3. As discussed in Section 6.2, the MRS is not able to predict movements based on the MRCP due to insufficient computing power. The provided times include the latency caused by the time required to compute the prediction according to Figure 6, Figure 9 and Figure 11. For a visualization of the prediction times relative to the movement onset see also Figure 2 and Figure 14. It can be observed that the modality and type of combination affects the prediction time.

For both the SRS and the FPGA-based system, the MRCP can predict upcoming movements earlier than the EMG (difference of means: SRS: 375 ms/FPGA: 406 ms, difference of medians: 442 ms/479 ms). The MoE provides a small increase of the prediction time in comparison to the MRCP (difference of means: 70/54 ms, difference of medians: 80/40 ms). However, if an and-based combination (MaE, PaMaE) is used, the prediction times are reduced.

### 6.6. Power Consumption

The power consumption for all systems and the signal combinations is shown in Table 4. For the FPGA-based system we evaluate the minimal configuration to process the desired physiological signals, i.e., the PL section of the Zynq contains only the DFHWAs required for the specific setup.

It can be observed that the FPGA-based system requires considerably less power than the SRS when idling, but also during computations. However, the power consumption is higher than the power consumption of the MRS. This effect is caused by the programmable logic: the power consumption increases proportional to the amount of utilized resources. Hence, when the system is equipped with multiple DFHWAs, the idle as well as computing power consumption increases.

For all systems, the power consumption increases when multiple physiological signals are processed. The highest power consumption of the SRS and MRS as well as the FPGA-based system is required if the MRCP and P300 detection as well as EMG processing are performed in parallel (i.e., the PaMaE and PaMoE combinations). Under these conditions the chosen LiFePO batteries (see Section 3.1) provide an operating time of approx. 3 h, which would be sufficient for most rehabilitation applications.

### 6.7. Final Hybrid System: Verification in Application

Based on the preliminary investigations (Section 6.2), 96 channels have been used for the MRCP and P300 configuration of the final hybrid system. Correspondingly, for EMG the highest classification performance and only a modest resource utilization is obtained for the channel combination ‘1∈[1,…,4]’, so that this channel setup is chosen. During the online evaluation we recorded the outputs of all single signals and their combinations. In addition, the raw data was recorded for a subsequent comparative offline analysis with a CPU-based system, which has been performed to obtain a reference for these datasets.

**Figure 15:**
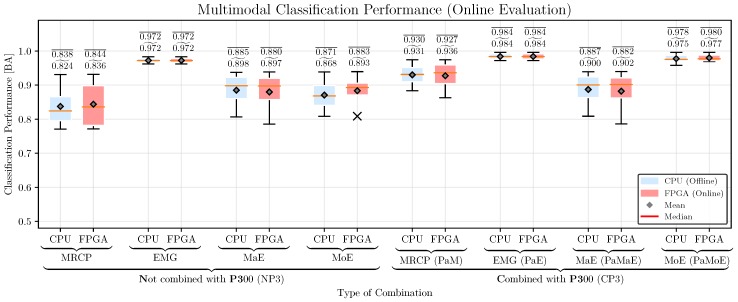
Classification Performance for Hybrid Movement Prediction for the Online Evaluation.

The classification performance obtained by using the FPGA-based system and the subsequent reference analysis with a CPU-based system is shown in Figure 15. We obtain a decreased classification performance in comparison to the offline evaluation (Figure 12) for the MRCP, but a similar performance for the EMG. The combination with other potentials can attenuate this reduction, i.e., we obtain a performance that is comparable to the offline performance. Similar to the offline analysis, we observe no difference in classification performance of the FPGA-based system in comparison to the CPU-based reference performance. Hence, the obtained results demonstrate that the proposed system can be used for online prediction under experimental conditions.

The overall resource utilization of the complete system is shown in Table 5. In addition to the PL resources required to implement the DFHWAs, resources needed to implement all auxiliary components (e.g., AXI bus system, DMA cores) are included in Table 5. The percentage values refer to the ZC7030 resources shown in Table 1. It can be observed that the resource utilization of the PL is less than 35% for all components. Hence, it would be possible to integrate further DFHWAs or other components into the system. Regarding the LUTs and FFs utilization of the complete system it can be observed that a significant proportion is required for the implementation of the auxiliary components, which is not the case for the BRAMs and DSP48 slices.

### 6.8. Summary

The obtained results show that the developed system can be used to predict upcoming movements before the actual physical movement onset. We confirm previous results from literature that an EEG-based movement prediction can predict movements earlier, but with a higher error rate than EMG [25]. For the investigated signals and signal combinations, we observed no significant difference in classification performance between the CPU- and FPGA-based systems in most cases. The parallel architecture of the system provides the possibility to process several streams of data in parallel and without the intervention of a CPU. This has two advantages. First, it provides the possibility to enhance the reliability of the system by combining the outputs from the MRCP and/or EMG-based DFHWAs. Second, it provides the possibility to choose the most suitable combination of predictions for the specific application. For instance, depending on the control algorithm of the rehabilitation device and the intended application, the EEG might be preferable since it can predict movements earlier in time, although it is less accurate than the EMG. Furthermore, in paralyzed or highly paretic patients caused by, e.g., spinal cord injury, the application of an EMG-based movement prediction might be infeasible.

The DFHWAs for all investigated signals provide significant speedups in comparison to the chosen mobile reference solution, but also in comparison to a standard PC. Furthermore, the system has a significantly decreased power consumption in comparison to the standard PC. Moreover, the resource utilization provides the possibility to integrate further algorithms in the same device, e.g., further DFHWAs for additional physiological signals and source localization methods to allow not only the prediction of upcoming movements, but also the affected extremity and/or movement direction [160,161]. Based on the provided computing performance, a future possibility would also be the possibility to perform movement prediction with an even higher time resolution than in the actual system, e.g., shift the segments for MRCP and EMG detection by 1 ms and apply a meta-classifier on the individual predictions.

Another advantage of our FPGA-based system is the possibility to establish a direct interface to other electronic devices. This makes it possible to control or adapt an assistive or rehabilitation device directly in real-time without the need of a software-based interface, which might introduce additional delays.

### 6.9. Comparison to Previous Work

Although combining ERD/ERS or MRCPs with EMG is a promising approach for the development of hybrid BCIs [42], there are still only few BCI systems that rely on this combination. Hence, the first part of the following comparison is limited to systems that combine ERD/ERS or MRCPs with EMG or other modalities. In the BCI systems listed in the first part of this comparison, the data processing is performed in standard personal computers or laptops, in contrast to out FPGA-based approach. However, as discussed in Section 1, neurorehabilitation applications would benefit from mobile or embedded BCI systems. Although FPGAs are well suited to build such systems (see Section 2.4 and Section 2.5), only few FPGA-based BCI systems exist. The second part of the comparison compares our system with FPGA-based BCIs.

Similar to our work, the combination of EEG and EMG for the detection of an arm has been investigated in [82]. Two different methods for the combination of EEG and EMG were investigated. It was shown that certain combinations obtain a better and more stable performance for the detection of movements in comparison to a single signal. Due to the EEG/EMG combination, the system can be used to compensate exhaustion or fatigue of the user. In contrast to our work, the subjects could move the left or right arm. However, the combination with further modalities, such as the P300, has not been investigated.

Multiple additional modalities next to EEG and EMG (e.g., eye gaze, Electrooculography (EOG), hand position) and their combinations to predict targets of human reaching motions were investigated in [84]. Similar to our work, it was shown that EEG predicts movements earlier that EMG, but with a lower accuracy than EMG. Furthermore, it was shown that EOG or eye tracking can also be used to improve the performance of EEG-based movement predictions. However, in contrast to our work, the study is limited to the combination of two modalities, a combination with the P300 has not been investigated.

In a previous work [25], we investigated the effect of combining EEG and EMG on the classification performance and reliability for the prediction of movements in an offline evaluation. However, in that work we neither considered the combination with further signals (i.e., the additional combination with P300), nor did we use a mobile FPGA-based system. Furthermore, we did not validate the approach in an online test.

In most other works, the combination of EEG and EMG is used for neurorehabilitation applications, e.g., to control orthoses or prostheses. For instance, in [83] it was shown that the realtime control of an upper limb wearable robot can be improved by such a combination. However, EEG was only used to compensate a lack of EMG, i.e., to estimate the torques of elbow and forearm based on EEG signals, if the EMG is has not enough power. The work shows that the torque estimation accuracy decreases if an EEG/EMG combination is used. The study does not investigate the effect of the combination on classification performance, if movements should be predicted.

The control of active orthoses for gait rehabilitation based on EEG and EMG has been presented in [43]. The work proposes an approach for the control of active orthoses that incorporates motion intention recognition based on the detection of ERD/ERS to predict the gait phase. In addition, EMG was used to estimate the active torque generated by the patient’s muscles. However, the control approach did also not consider the fusion of EEG and EMG to enhance the classification accuracy to predict movements.

A hybrid control approach for an upper limb prosthesis based on EEG and EMG was investigated in [86]. EEG was used to control the exoskeleton asynchronously, joint angles were estimated using EMG. The study shows that it is possible to estimate the joint angles with root-mean-square errors of less than $6^{\circ }$. However, similar to the studies discussed above, it was not investigated whether a combination of EEG and EMG can be used to predict movements with an improved accuracy.

A combined EEG/EMG classification for hand and wrist movements in transhumeral amputees was investigated in an offline study in [87]. It was shown that the EEG/EMG combination outperforms the classification by either EEG or EMG. However, in contrast to the study in this paper, the aim was to differentiate between different movements instead of predicting upcoming movements. The authors point out that a miniature and portable system is required for future practical applications.

In the literature discussed above, the data processing was based on standard PCs. The aim of FPGA-based BCI systems is to replace a standard PC. In [107,109], Shyu et. al. present an FPGA-based system for the detection of SSVEPs. The system can be used for the control of a multimedia system [107] or a hospital bed [109]. Especially the tight integration of EEG signal processing and motor control to actuate the hospital bed represents an interesting opportunity for other rehabilitation devices. However, since these systems target the detection of SSVEPs, they have been designed to process only two EEG channels, and are hence not usable for the detection of, e.g., MRCPs.

The work in [108] presents a low-cost FPGA-based P300 speller. Similar to us, they achieve a classification performance that is comparable to a standard CPU. However, most parts of the system are implemented using softcore CPUs (only a band-pass Butterworth filter is implemented in hardware), and only seven EEG channels can be used. However, the aim of the system is to detect the P300, the detection of ERD/ERS or MRCP is not considered.

A recent work uses an FPGA-based prototype for fall prediction in everyday life [110], which is based on a previous software-implementation [85]. Similar to our work they combine EEG processing (for the detection of movement related potentials) with EMG and show in an offline evaluation that it is possible to obtain a small numerical error, despite the usage of fixed-point arithmetic in the FPGA. However, for the fall prediction, a fixed number of only 7 EEG and 8 EMG channels is sufficient; the classification performance, resource utilization and computing time for different numbers of channels has not been investigated. The proposed system requires 56 ms for the prediction of a movement, which is within the reported application time limit of 300 ms for fall prediction, while the MRCP prediction of our system requires approx. 0.95 ms for the prediction of a movement using 124 EEG channels (see Figure 9). The study does not investigate the effect of different combination methods on the classification performance.

In previous works [162,163], we presented preliminary systems for the detection of the P300 and MRCPs, respectively, using FPGA-based systems. However, these systems were inflexible and did not exploit the full parallel processing capabilities to process multiple physiological signals at the same time, so that these systems were only capable to detect either the P300 or MRCP. These systems did not support the fusion of multiple signals, which can improve the classification performance significantly, as shown in this work. Furthermore, these preliminary systems have not been validated in an online evaluation.

Overall, this comparison shows that our FPGA-based system has, similar to other recent hybrid BCI systems, the capability to process EEG and EMG data in combination. However, to the best of our knowledge, the combination of MRCP, EMG and additionally the P300 has not been addressed before. Furthermore, the capabilities of our FPGA-based system can be useful in applications that require miniaturized BCI-systems with high requirements on performance and flexibility that cannot be fulfilled by other previously proposed FPGA-based BCI-systems. Hence, it can help to improve the capabilities of future neurorehabilitation devices.

### 6.10. Limitations

The evaluation of the proposed FPGA-based system shows that it is feasible to use it for movement prediction using EEG and EMG data. However, several limitations of the study should be noted.

First, the study has been conducted under laboratory conditions. Typical rehabilitation applications will be performed under more uncontrolled conditions. Thus, the EEG and EMG will be affected by artifacts and different kinds of noise. We have chosen the present study design to provide the possibility to determine the time of the movement onset exactly and to be able to compare the obtained results to other studies [25,64]. However, the methodology for the detection of the MRCP and P300 has already been successfully applied in other applications, e.g., the usage of MRCP-based movement prediction to enhance the user-experience of an exoskeleton by adapting the control algorithms [58] and the P300 detection for the monitoring of the user’s state [57]. Hence, since we also observe no significant performance difference between the CPU and FPGA-based implementations, we assume that the developed system will work under such conditions without major modifications.

Secondly, the current study was conducted using healthy subjects. Although it has been shown that patients that suffer from, e.g., stroke, are feasible to perform movement-imagination despite chronic or severe motor impairments [68,69,164], different kinds of brain damages can affect the EEG [165,166,167,168,169,170] and EMG [171,172], and hence, possibly the performance of the proposed system. However, MRCP-based BCIs can be successfully used by patients suffering from stroke [173,174,175]. For other neurological diseases, such as amyotrophic lateral sclerosis, it has been shown that certain MRCP characteristics do not differ from healthy patients [176]. Hence, we assume that the proposed system can be used by patients with neurological motor impairments. Since the system supports different types of signal combinations for movement prediction, it is possible to choose an operation mode depending on the specific conditions of a patient. Nevertheless, the specific difference in classification performance between healthy and impaired subjects depending on impairment and operation mode has to be investigated in the future.

Thirdly, there are mobile systems available with more advanced CPU architectures and higher clock frequencies than the MRS that was used in the experiments for comparison. However, although these might provide a higher computational performance than the MRS, they are less extensible than the proposed FPGA-based system. Due to the low resource utilization of the DFHWAs, we are able to integrate further features like artifact removal methods, eye-tracking [177], physiological signals to monitor fatigue or exhaustion, and also control algorithms and motor commutation components to drive a rehabilitation device directly.

## 7. Conclusions and Future Directions

In this paper, we presented a novel mobile system based on reconfigurable hardware and dataflow computing for the online processing of multiple streams physiological data. The system provides the capability to predict movements based on an online analysis of multiple streams of data in parallel. This can be used to generate hybrid predictions by combining EEG and EMG-based movement predictions or movement predictions with task-related patterns, i.e., the P300. We showed that the hybrid movement prediction can result in a significantly improved classification performance over single predictions. The developed system has been evaluated in a study that consists of 12 subjects in total. It is able to outperform standard CPU-systems regarding prediction time and provides a lower power consumption than a standard PC. Furthermore, we showed that the fixed-point arithmetic that was used in the FPGA-implementation does not result in a decrease of the classification performance.

The next steps are the integration of the proposed system into an exoskeleton for robotic rehabilitation scenarios [12] and the combination of the control algorithms of the exoskeleton with the movement prediction in a single SoC design. Such a tight integration will allow a real-time adaptation of the control algorithms of the exoskeleton to provide the possibility of an assist-as-needed rehabilitation [29]. Furthermore, in a future study we will evaluate the applicability of the system for patients suffering from movement impairments due to stroke and extend its capabilities with additional features to remove artifacts and monitor the exhaustion of patients.

## Figures and Tables

**Table 1:** Programmable logic resources of the Xilinx ZC7030.

Programmable	Look-Up Tables	Flip-Flops	BRAM	DSP Slices
Logic Cells	(LUT)	(FF)	(36 kb Each)	(DSP48)
125,000	78,600	157,200	265	400

**Table 2:** Mean False Negative Rate (FNR), False Positive Rate (FPR = 1-Recall) and Precision for the Hybrid Movement Prediction.

	Not Combined with P300 (NP3)								Combined with P300 (CP3)							
	**MRCP**		**EMG**		**MaE**		**MoE**		**MRCP**		**EMG**		**MaE**		**MoE**	
	**CPU**	**FPGA**	**CPU**	**FPGA**	**CPU**	**FPGA**	**CPU**	**FPGA**	**CPU**	**FPGA**	**CPU**	**FPGA**	**CPU**	**FPGA**	**CPU**	**FPGA**
Mean FNR	0.104	0.116	0.063	0.063	0.278	0.290	0.021	0.019	0.116	0.127	0.063	0.062	0.282	0.293	0.021	0.020
Mean FPR	0.147	0.142	0.029	0.034	0.006	0.007	0.169	0.168	0.022	0.021	0.005	0.006	0.002	0.002	0.033	0.033
Mean Precision	0.108	0.109	0.332	0.303	0.577	0.541	0.123	0.121	0.422	0.421	0.711	0.695	0.826	0.810	0.403	0.408

**Table 3:** Prediction times in ms *before* movement onset (N/A: not available).

Movement Prediction Times																
	MRCP				EMG				MaE				MoE			
System	$\mu_{t}$	$Q_{t}^{75}$	$Q_{t}^{50}$	$Q_{t}^{25}$	$\mu_{t}$	$Q_{t}^{75}$	$Q_{t}^{50}$	$Q_{t}^{25}$	$\mu_{t}$	$Q_{t}^{75}$	$Q_{t}^{50}$	$Q_{t}^{25}$	$\mu_{t}$	$Q_{t}^{75}$	$Q_{t}^{50}$	$Q_{t}^{25}$
SRS	515	234	514	834	140	39	79	199	124	34	74	154	585	274	594	914
SRS (2)	516	235	515	835	140	39	79	199	125	35	75	155	586	275	595	915
SRS (4)	516	235	515	835	140	39	79	199	125	35	75	155	586	275	595	915
MRS	N/A	N/A			134	33	73	193	N/A	N/A			N/A	N/A		
MRS (2)	N/A	N/A			134	33	73	193	N/A	N/A			N/A	N/A		
FPGA	542	239	559	879	136	40	80	200	129	39	79	159	596	269	599	919
	PaM				PaE				PaMaE				PaMoE			
System	$\mu_{t}$	$Q_{t}^{75}$	$Q_{t}^{50}$	$Q_{t}^{25}$	$\mu_{t}$	$Q_{t}^{75}$	$Q_{t}^{50}$	$Q_{t}^{25}$	$\mu_{t}$	$Q_{t}^{75}$	$Q_{t}^{50}$	$Q_{t}^{25}$	$\mu_{t}$	$Q_{t}^{75}$	$Q_{t}^{50}$	$Q_{t}^{25}$
SRS	314	194	234	354	125	34	74	194	190	34	74	154	340	194	234	394
SRS (2)	315	195	235	355	126	35	75	195	191	35	75	155	340	195	235	395
SRS (4)	315	195	235	355	126	35	75	195	191	35	75	155	341	195	235	395
MRS	N/A	N/A			N/A	N/A			N/A	N/A			N/A	N/A		
MRS (2)	N/A	N/A			N/A	N/A			N/A	N/A			N/A	N/A		
FPGA	319	199	239	359	129	39	79	199	199	39	79	159	355	199	239	399

**Table 4:** Power consumption, reference versus FPGA-based system with different configurations.

**SRS**	**Idle**	**1 Core**	**2 Cores**	**4 Cores**
EMG	119.8 W	130.2 W	130.2 W	130.2 W
MRCP	119.8 W	126.0 W	126.4 W	127.6 W
MaE, MoE	119.8 W	133.0 W	133.3 W	135.2 W
PaM	119.8 W	129.6 W	130.2 W	132.1 W
PaE	119.8 W	132.6 W	132.6 W	132.6 W
PaMaE, PaMoE	119.8 W	136.8 W	138.1 W	138.3 W
**MRS**	**Idle**	**1 Core**	**2 Cores**	
EMG	2.98 W	3.28 W	3.23 W	
MRCP	2.98 W	3.23 W	3.23 W	
MaE, MoE	2.98 W	3.30 W	3.61 W	
PaM	2.98 W	3.29 W	3.60 W	
PaE	2.98 W	3.31 W	3.64 W	
PaMaE, PaMoE	2.98 W	3.32 W	3.66 W	
**FPGA**	**Idle**	**Computing**		
EMG	3.45 W	4.12 W		
MRCP	3.45 W	4.12 W		
MaE, MoE	3.49 W	4.41 W		
PaM	3.58 W	4.48 W		
PaE	3.53 W	4.49 W		
PaMaE, PaMoE	3.59 W	4.51 W		

**Table 5:** Resource utilization for the complete system.

Type of Resource	LUT	FF	36 kb Each	DSP48
DFHWA	15 755 (20.0%)	16 786 (10.7%)	53.5 (20.1%)	136 (34%)
Auxiliary Components	6 084 (7.7%)	7 529 (4.8%)	7.5 (2.8%)	0 (0%)
Total	21 839 (27.8%)	24 315 (15.4%)	61 (23.0%)	136 (34%)

## Supplementary Materials

The following are available online at http://www.mdpi.com/1424-8220/17/7/1552/s1.

## Abbreviations

**ASIC**	Application-Specific Integrated Circuit
**BA**	Balanced Accuracy
**BCI**	Brain-Computer Interface
**BRAM**	Block Random Access Memory
**CPU**	Central Processing Unit
**DFHWA**	Dataflow Hardware Accelerator
**DMA**	Direct Memory Access
**DSP**	Digital Signal Processing
**EMG**	Electromyography
**EOG**	Electrooculography
**ERD/ERS**	Event-Related Desynchronization/Synchronization
**EEG**	Electroencephalography
**ERP**	Event-Related Potential
**FIFO**	First-In, First-Out
**FIR**	Finite Impulse Response
**FF**	Flip Flop
**FNR**	False Negative Rate
**FPGA**	Field Programmable Gate Array
**FPR**	False Positive Rate
**GPU**	Graphics Processing Unit
**IIR**	Infinite Impulse Response
**ISI**	Inter-Stimulus Interval
**IRQ**	Interrupt ReQuest
**LUT**	Look-Up Table
**MAC**	Multiply ACcumulate
**ML**	Machine Learning
**MRCP**	Movement Related Cortical Potential
**MRS**	Mobile CPU-based Reference System
**PE**	Processing Element
**PL**	Programmable Logic
**PS**	Processing System
**SDF**	Synchronous Dataflow
**SoC**	System-on-Chip
**SSVEP**	Steady-State Visual Evoked Potential
**SNR**	Signal-to-Noise Ratio
**SVM**	Support Vector Machine
**SRS**	Standard-PC Reference System
**TPR**	True Positive Rate
**TNR**	True Negative Rate
