# Supplementary material for: A Hybrid FPGA-Based System for EEG- and EMG-Based Online Movement Prediction
Source: Sensors (Basel). 2017 Jul 3;17(7):1552. doi: 10.3390/s17071552 (PMC5539567; doi:10.3390/s17071552)
Supplement: Supplementary file 1 [file sensors-17-01552-s001.pdf]

Supplementary Materials: A Hybrid FPGA-Based System for EEG- and EMG-Based Online Movement Prediction

Hendrik Wöhrle, Marc Tabie, Su Kyoung Kim, Frank Kirchner and Elsa Andrea Kirchner

1. Performance Scores

This supplement contains supplementary performance scores (precision, recall and specifity) for the results shown in the main manuscript.

1.1. Supplementary performance scores for the EMG-based movement prediction

Table S1. Precision, Recall and Specificity for the EMG-based movement prediction, channels 1 to 4.

Channel	1		2		3		4	
**System type**	**CPU**	**FPGA**	**CPU**	**FPGA**	**CPU**	**FPGA**	**CPU**	**FPGA**
Mean Precision	0.278	0.280	0.303	0.303	0.102	0.102	0.132	0.119
Median Precision	0.272	0.272	0.309	0.309	0.106	0.106	0.106	0.099
Mean Recall/TPR	0.808	0.808	0.841	0.841	0.714	0.714	0.847	0.854
Median Recall/TPR	0.879	0.879	0.947	0.947	0.707	0.707	0.882	0.905
Mean Specifity/TNR	0.974	0.974	0.975	0.975	0.921	0.921	0.907	0.895
Median Specifity/TNR	0.973	0.973	0.973	0.973	0.923	0.923	0.926	0.904

Table S2. Precision, Recall and Specificity for the EMG-based movement prediction, channels 5 to 8.

Channel	5		6		7		8	
**System type**	**CPU**	**FPGA**	**CPU**	**FPGA**	**CPU**	**FPGA**	**CPU**	**FPGA**
Mean Precision	0.020	0.021	0.017	0.017	0.016	0.016	0.018	0.018
Median Precision	0.013	0.012	0.015	0.016	0.014	0.014	0.016	0.016
Mean Recall/TPR	0.071	0.070	0.250	0.250	0.237	0.237	0.323	0.323
Median Recall/TPR	0.049	0.041	0.240	0.240	0.195	0.195	0.316	0.316
Mean Specifity/TNR	0.955	0.956	0.854	0.855	0.863	0.863	0.813	0.813
Median Specifity/TNR	0.965	0.966	0.844	0.845	0.862	0.863	0.806	0.805

Table S3. Precision, Recall and Specificity for the EMG-based movement prediction, for channel combinations 1 ∈ [1,…,8] to 2 ∈ [1,…,4].

Channel	1 ∈ [1,…,8]		2 ∈ [1,…,8]		4 ∈ [1,…,8]		1 ∈ [1,…,4]		2 ∈ [1,…,4]	
**System type**	**CPU**	**FPGA**	**CPU**	**FPGA**	**CPU**	**FPGA**	**CPU**	**FPGA**	**CPU**	**FPGA**
Mean Precision	0.210	0.206	0.209	0.122	0.198	0.198	0.332	0.303	0.412	0.168
Median Precision	0.195	0.191	0.164	0.112	0.149	0.148	0.319	0.296	0.455	0.163
Mean Recall/TPR	0.931	0.908	0.851	0.946	0.485	0.496	0.937	0.937	0.882	0.964
Median Recall/TPR	0.961	0.929	0.899	0.972	0.398	0.393	0.978	0.978	0.950	0.985
Mean Specifity/TNR	0.947	0.947	0.945	0.898	0.963	0.963	0.971	0.966	0.982	0.928
Median Specifity/TNR	0.946	0.946	0.957	0.900	0.980	0.980	0.973	0.971	0.987	0.933

1.2. Supplementary performance scores for the MRCP-based movement prediction

Table S4. Precision, Recall and Specificity for the MRCP-based movement prediction.

**Number of channels**	**32**		**64**		**96**		**124**	
**System type**	**CPU**	**FPGA**	**CPU**	**FPGA**	**CPU**	**FPGA**	**CPU**	**FPGA**
Mean Precision	0.084	0.081	0.095	0.092	0.108	0.109	0.116	0.111
Median Precision	0.086	0.091	0.101	0.099	0.106	0.109	0.114	0.101
Mean Recall/TPR	0.887	0.856	0.891	0.878	0.896	0.884	0.867	0.866
Median Recall/TPR	0.914	0.894	0.911	0.897	0.925	0.912	0.909	0.877
Mean Specifity/TNR	0.776	0.808	0.821	0.818	0.853	0.858	0.868	0.863
Median Specifity/TNR	0.776	0.808	0.821	0.818	0.853	0.858	0.868	0.863

1.3. Supplementary performance scores for the P300 detection

Table S5. Precision, Recall and Specificity for the P300 detection.

**Number of channels**	**32**		**64**		**96**		**124**	
**System type**	**CPU**	**FPGA**	**CPU**	**FPGA**	**CPU**	**FPGA**	**CPU**	**FPGA**
Mean Precision	0.695	0.710	0.736	0.739	0.750	0.750	0.776	0.776
Median Precision	0.713	0.734	0.744	0.764	0.773	0.779	0.783	0.772
Mean Recall/TPR	0.869	0.871	0.868	0.871	0.858	0.877	0.861	0.860
Median Recall/TPR	0.882	0.872	0.859	0.857	0.860	0.880	0.868	0.869
Mean Specifity/TNR	0.951	0.954	0.961	0.960	0.962	0.962	0.969	0.969
Median Specifity/TNR	0.955	0.962	0.963	0.967	0.967	0.970	0.975	0.971
